# Repurposing drugs for the prevention of vascular dementia using evidence from drug target Mendelian randomization

**DOI:** 10.1038/s43587-026-01106-1

**Published:** 2026-04-20

**Authors:** Victoria Taylor-Bateman, Phazha Bothongo, Venexia Walker, Patrick G. Kehoe, Liv Tybjærg Nordestgaard, Yoav Ben-Shlomo, Neil M. Davies, Dylan M. Williams, Emma L. Anderson

**Affiliations:** 1 Division of Psychiatry, University College London, London, UK; 2 Medical Research Council Integrative Epidemiology Unit, Bristol Medical School, University of Bristol, Bristol, UK; 3 Department of Surgery, Perelman School of Medicine, University of Pennsylvania, Philadelphia, PA USA; 4 Cerebrovascular and Dementia Research Group, Bristol Medical School, University of Bristol, Learning & Research, Southmead Hospital, Bristol, UK; 5 Department of Clinical Biochemistry, Copenhagen University Hospital – Bispebjerg and Frederiksberg, Copenhagen, Denmark; 6 Population Health Sciences, Bristol Medical School, University of Bristol, Bristol, UK; 7 Department of Statistical Science, University College London, London, UK; 8 Department of Public Health and Nursing, Norwegian University of Science and Technology, Trondheim, Norway; 9 Unit for lifelong health and ageing, University College London, London, UK

**Keywords:** Dementia, Ageing, Drug discovery, Genetics

## Abstract

Vascular dementia (VaD) is a devastating cerebrovascular disease with no disease-modifying treatments. Repurposing drugs for known risk factors could have clinical impact. Using Mendelian randomization, we proxied 46 lipid-lowering, antihypertensive and anti-inflammatory drug effects across five VaD outcomes: clinical diagnosis (*N* = 7,009 cases, *N* = 899,672 non-cases/controls) and neuroimaging features (max *N* = 50,559), white matter hyperintensity volume, fractional anisotropy, mean diffusivity and lacunar stroke diagnosis. Beta-1 adrenergic receptor indicated potential benefit (clinical diagnosis: odds ratio (OR) = 0.90, 95% confidence interval (CI) = 0.80–1.01; white matter hyperintensity volume: estimated causal effect = −0.03, 95% CI = −0.07–0.00; mean diffusivity: estimated causal effect = −0.18, 95% CI = −0.37–0.00; lacunar stroke: OR = 0.91, 95% CI = 0.80–1.03). Angiotensin-converting enzyme inhibition suggested increased VaD risk (OR = 1.12, 95% CI = 1.01–1.24). Findings remained largely null after multiple-testing correction. Here we show that although little evidence supported repurposing most lipid-lowering, antihypertensive and anti-inflammatory drugs for VaD prevention or treatment, beta-1 adrenergic receptor antagonism could be a promising repurposing candidate, but replication is needed as further data becomes available. Pharmacovigilance studies should examine angiotensin-converting enzyme inhibitors’ potential to increase risk.

## Abstract (web summary)

Repurposing existing drugs for vascular dementia (VaD) could have clinical impact. Here, using Mendelian randomization, researchers tested whether cholesterol, anti-inflammatory or blood pressure drug targets affect risk of VaD, finding little evidence to support repurposing opportunities. A potential benefit was seen for beta-blocker target ADRB1 while ACE inhibition was observed to potentially increase risk of VaD.

## Main

More than 55 million people are living with dementia globally, but very few effective treatments are available. Evidence of cerebrovascular disease is present in almost 80% of all dementia cases^1^; and there is some evidence that it might be one of the earliest pathological changes that occurs in Alzheimer’s disease^2^, which is still widely reported as the leading cause of dementia. Yet vascular dementia (VaD) has received comparatively little attention and research funding^3^. VaD is a progressive cerebrovascular disease caused by brain injury as a result of impaired cerebral blood flow^4^. There are currently no effective treatments or preventive therapeutics for VaD; options are limited to modifying risk factors such as cholesterol levels and blood pressure and managing symptoms. Drug repurposing can reduce both the time and cost associated with introducing new treatments while leveraging established safety profiles and existing regulatory approvals. It has been widely explored in the case of Alzheimer’s disease, with more than 573 drugs proposed as therapeutic candidates in the last 10 years^5^. However, research into drug repurposing for VaD is scarce^6–9^, and current findings are limited. In addition to most drug trials being very small (*n* < 500), they have also been restricted to participants of old age (for example >70 years). This is problematic because the onset of prodromal cerebrovascular pathology begins in midlife^10^, and any preventative interventions are likely to be more effective when administered as early as possible in the disease course. Yet conducting clinical trials in which preventative drugs are initiated in midlife, with sufficiently long clinical follow-up periods to detect VaD outcomes, is not feasible or cost-effective.

Genetic epidemiology provides another source of evidence about the potential effects of licensed and new drugs, and evidence has shown that therapeutics with human genetic support are more than twice as likely to achieve regulatory approval than agents lacking genetic support^11^. Drug target Mendelian randomization (MR) is an instrumental variable (IV) method that can be used to identify potential high-priority therapeutic targets^12^. It has already been successfully applied in other disease areas: for example, to support the repurposing of interleukin-6 receptor inhibitors to treat severe COVID-19 infection^13,14^. In drug target MR, the expression or function of protein drug targets are instrumented by genetic variants within or near the genes that encode them. These genetic effects are then used to estimate the effects of the drug target on a disease or proxy endpoint. Genotypes are inherited randomly during conception, analogous to random treatment allocation in randomized trials. Thus, associations of genetic markers for both drug targets and disease outcomes are unlikely to be biased by confounding or reverse causation, which limits causal inference in traditional observational epidemiology methods. With the wealth of publicly available genetic data, this method represents an efficient and cost-effective approach to identifying druggable targets or evaluating the repurposing potential of existing drugs.

Prior studies have suggested that blood pressure and low-density lipoprotein cholesterol (LDL-c) are causal risk factors for VaD^15^. Systemic inflammation is also thought to be an important part of the disease’s pathogenesis, with higher C-reactive protein (CRP) levels having been previously associated with VaD neuroimaging markers^16^. This study aimed to evaluate the potential for repurposing a range of antihypertensive, lipid-lowering and anti-inflammatory drugs to reduce risk of VaD using two-sample drug target MR. A total of five outcome datasets for VaD were used: VaD diagnosis, white matter hyperintensity (WMH) volume, inversed fractional anisotropy (iFA), mean diffusivity (MD) and lacunar stroke (LS).

## Results

**Fig. 1:**
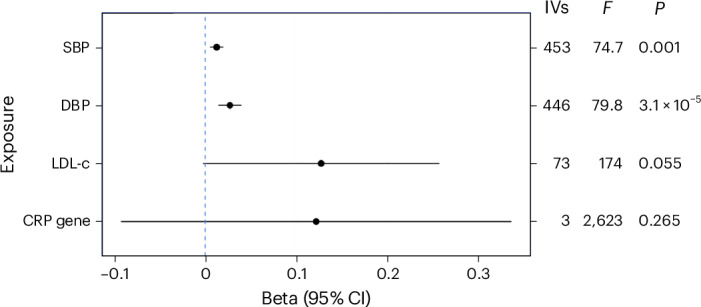
Initial downstream biomarker MR comparison with VaD. IVW MR comparison using European genome-wide association data. Each of the four downstream biomarker datasets were used as the exposure with VaD (VaD diagnosis, *n* = 7,008/899,672 cases/controls) as the outcome. The four exposures were SBP (*n* = 757,601), DBP (*n* = 757,601), LDL-c, *n* = 188,578) and CRP, *n* = 575,531). Analysis for CRP was restricted to its gene region; the remaining exposures had instruments selected from the entire genome. Data are presented as the causal MR estimate as the center and error bars indicating a ±95% CI, with the minima corresponding to the lower 95% CI bound and the maxima representing the upper 95% CI bound. The number of IVs used in each analysis is given as well as the *P* values. The *F*-statistic is also provided as a measure of instrument strength.

Of the 46 drug targets identified, suitable instruments were identified for 38 targets in the downstream biomarker genome-wide association study (GWAS) data (all gene regions except ACLY, ALOX5, ATIC, KCNMA1, MTTP, NR3C1, TLR7 and TNF; Table 1). Where an analysis was not performed, this was due to either data not being present or a lack of suitable instruments meeting either genome-wide threshold (*P* < 5 × 10^−8^) or a reduced *P*-value threshold of *P* < 5 × 10^−5^. As expected, the initial downstream biomarker MR comparison results show that increasing diastolic blood pressure (DBP), systolic blood pressure (SBP) and LDL-c levels are associated with increased risk of VaD (DBP: OR = 1.03, CI = 1.01–1.04, SBP: OR = 1.01, CI = 1.01–1.02, LDL-c: OR = 1.14, CI = 0.997–1.29). The results for CRP were consistent in direction and similar in magnitude to LDL-c but lacked precision (OR = 1.13, CI = 0.91–1.40) (Fig. 1 and Supplementary Table 1).

**Table 1:** A list of the identified drug targets by class and which analysis was performed

Class	Example drug	Target	Type	Action	Main analysis:	Sensitivity analysis:
					Downstream biomarker	pQTL
**Lipid-lowering drugs**						
ACL inhibitor	Bempedoic acid	ACLY	Inhibitor	−ve	x	x
ANGPTL inhibitors	Evinacumab	ANGPTL3	Inhibitor	−ve	✓	✓
ApoB antisense	Mipomersen	APOB	Inhibitor	−ve	✓	✓
MTTP inhibitors	Lomitapide	MTTP	Antagonist	−ve	x	x
NPC1L1 blocker	Ezetimibe	NPC1L1	Inhibitor	−ve	✓	x
PCSK9 inhibitors	Alirocumab	PCSK9	Inhibitor	−ve	✓	✓
Statins	Simvastatin	HMGCR	Inhibitor	−ve	✓	x
**Antihypertensive drugs**						
ACE inhibitors	Catopril	ACE	Inhibitor	−ve	✓	✓
Aldosterone receptor antagonist	Spironolactone	NR3C2	Antagonist	−ve	✓	x
Alpha-2 adrenergic agonists	Methyldopa	ADRA2A	Agonist	+ve	✓*	x
Alpha-blockers	Doxazosin	ADRA1A	Antagonist	−ve	✓	x
	Doxazosin	ADRA1D	Antagonist	−ve	✓	x
Angiotensin receptor antagonists	Losartan	AGTR1	Antagonist	−ve	✓*	x
Beta-blockers	Metoprolol	ADRB1	Antagonist	−ve	✓	x
Calcium channel blockers	Verapamil	CACNA1C	Inhibitor	−ve	✓	✓*
Loop diuretics	Furosemide	SLC12A1	Inhibitor	−ve	✓*	x
Renin inhibitors	Aliskiren	REN	Inhibitor	−ve	✓	✓
Thiazide diuretics	Hydrochlorothiazide	SLC12A3	Inhibitor	−ve	✓	x
	Hydrochlorothiazide	KCNMA1	Inhibitor	−ve	x	x
Vasodilators	Minoxidil	KCNJ1	Inducer	+ve	✓	x
**Anti-inflammatories**						
COX inhibitors	Naproxen	PTGS1	Inhibitor	−ve	✓*	x
	Naproxen	PTGS2	Inhibitor	−ve	✓*	x
Glucocorticoids	Prednisolone	NR3C1	Agonist	+ve	x	x
csDMARDs	Methotrexate	TYMS	Inhibitor	−ve	✓*	✓*
	Methotrexate	ATIC	Inhibitor	−ve	x	x
	Methotrexate	DHFR	Inhibitor	−ve	✓*	✓*
csDMARDs	Hydroxychloroquine	TLR7	Antagonist	−ve	x	x
		TLR9	Antagonist	−ve	✓*	x
csDMARDs	Leflunomide	DHODH	Inhibitor	−ve	✓*	x
csDMARDs	Sulfasalazine	ALOX5	Inhibitor	−ve	x	x
		ACAT1	Inhibitor	−ve	✓*	✓
		PLA2G1B	Antagonist	−ve	✓*	✓*
		IKBKB	Inhibitor	−ve	✓	x
csDMARDs	Ciclosporin	CAMLG	Binder	−ve	✓	x
		PPP3R2	Inhibitor	−ve	✓	x
		PPIA	Inhibitor	−ve	✓	✓*
		PPIF	Binder	−ve	✓	✓*
TNF inhibitors	Adalimumab	TNF	Inhibitor	−ve	x	✓
IL-6 inhibitors	Tocilizumab	IL6R	Inhibitor	−ve	✓	✓
bsDMARDs	Rituximab	MS4A1	Binder		✓	x
bsDMARDs	Abatacept	CD80	Antagonist	−ve	✓	✓
	Abatacept	CD86	Antagonist	−ve	✓*	✓
IL-1 inhibitors	Anakinra	IL1R1	Inhibitor	−ve	✓	✓
Janus kinase inhibitors	Tofacitinib	JAK1	Inhibitor	−ve	✓	x
	Tofacitinib	JAK2	Inhibitor	−ve	✓*	✓
	Tofacitinib	JAK3	Inhibitor	−ve	✓*	x

A summary of the drug targets chosen for analysis, with an example of an existing drug targeting the protein and the type of action of that drug. A list of analyses performed for each target is also given as indicated (✓) for the primary analysis method using downstream biomarker gene flanking, and the additional sensitivity analysis method using *cis*-acting pQTL analysis. A cross (x) indicates the analysis could not be performed. Targets with IVs that met only a reduced *P*-value threshold (5 × 10^−5^) are indicated with a (*). Effect directionality: −ve, negative; +ve, positive.

### Lipid-lowering targets

**Fig. 2:**
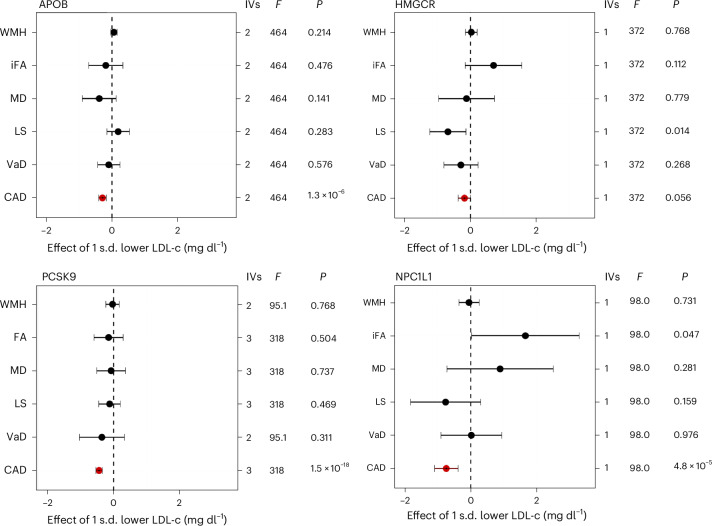
Drug target MR for lipid-lowering drug targets. IVW MR was conducted for lipid-lowering drug targets using large-scale European GWAS summary statistics. The *cis*-acting gene region was selected for each target with LDL-c (a downstream biomarker) GWAS used as the exposure (*n* = 188,578), with five VaD outcomes: WMH (*n* = 50559), iFA, *n* = 31,125), MD (*n* = 31,147), LS (*n* = 6,030/248,929 cases/controls), VaD (VaD diagnosis, *n* = 7,008/899,672 cases/controls). CAD was used as a positive control and is indicated in red (*n* = 122,733/424,528 cases/controls). Data are presented as the causal MR estimate as the center and error bars indicating a ± a 95% CI, with the minima corresponding to the lower 95% CI bound and the maxima representing the upper 95% CI bound. The number of IVs used in each analysis is given: for targets with multiple IVs, IVW MR was used; for those with a single IV, the Wald ratio was calculated. The *F*-statistic is also provided as a measure of instrument strength. *P* values are uncorrected; multiple-comparison corrected *P* values (applied at differing levels) are available in the Supplementary Information.

**Extended Data Fig. 1:**
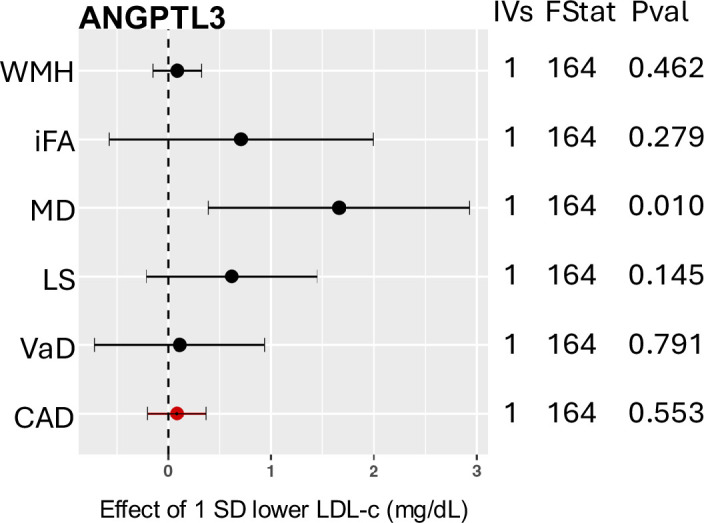
Additional Plots for Lipid Lowering Drug Target MR using LDL Downstream Biomarker Data, for which there was no reductive effect on the positive control. Additional plots for inverse variance-weighted MR (Mendelian randomization) for lipid-lowering drug targets using large-scale European genome-wide association study (GWAS) summary statistics. The cis-acting gene region was selected for each target from a LDL-c (a downstream biomarker) GWAS (n = 188578) as the exposure with five vascular dementia outcomes; WMH (white matter hyperintensities, n = 50559), iFA (inversed fractional anisotropy, n = 31125), MD (mean diffusivity, n = 31147), LS (lacunar stroke, n = 6030/248929 cases/controls), VaD (vascular dementia diagnosis, n = 7008/899672 cases/controls). CAD (coronary artery disease) was used as a positive control (n = 122733/424528 cases/controls) and is indicated in red. The additional target presented here did not show the expected lowering effect on the positive control (Pval>0.1) and as only a single IV was used for analysis a Wald ratio was calculated. Data are presented as the causal MR estimate as the centre and error bars indicating a +/- a 95% confidence interval (CI), with the minima corresponding to the lower 95% CI bound and the maxima representing the upper 95% CI bound. The number of instrumental variables (IVs) used in each analysis is given. The F-statistic (Fstat) is also provided as a measure of instrument strength. Pval denotes uncorrected p-values; multiple-comparison corrected p-values (applied at differing levels) are available in the supplement. LDL-c (low-density lipoprotein cholesterol), SD (standard deviation).

Figure 2 shows the results for lipid-lowering targets for which there was evidence of an effect in the expected direction on the positive control, coronary artery disease (CAD). Targets without the expected effect on the positive control can be found in Extended Data Fig. 1; full results for all targets are in Supplementary Table 2. Overall, there was little consistent evidence suggesting that lipid-lowering drug targets had a causal effect on VaD. There was evidence that HMGCR inhibition may reduce the risk of LS (OR = 0.50, CI = 0.29–0.87) and weak evidence that it may reduce VaD risk. However, the VaD estimate lacked precision, despite the point estimate being larger than that for HMGCR on CAD. NPC1L1 reduction was associated with increased iFA (*β* = 1.65, CI = 0.02–3.28). There was little evidence for the effect of these targets on any other outcomes.

### Antihypertensive targets

**Fig. 3:**
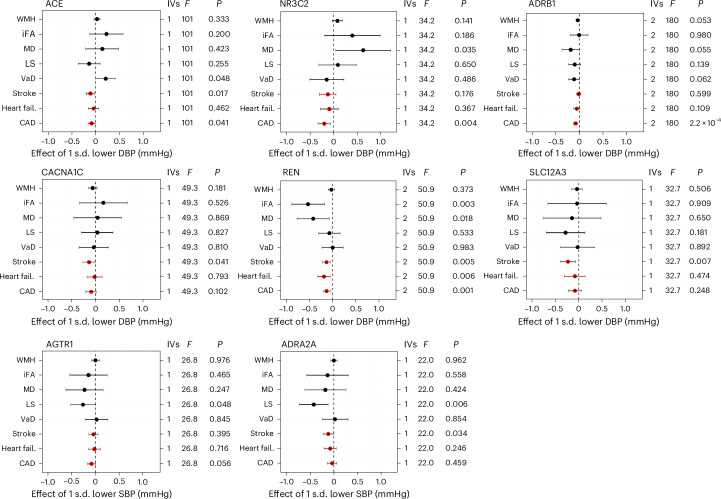
Drug target MR for antihypertensive drug targets. MR or was conducted for antihypertensive drug targets using large-scale European GWAS summary statistics. The *cis*-acting gene region was selected for each target using blood pressure (a downstream biomarker) GWAS as the exposure with five VaD outcomes: WMH (*n* = 50,559), iFA (*n* = 31,125), MD (*n* = 31,147), LS (*n* = 6,030/248,929 cases/controls), VaD (VaD diagnosis, *n* = 7,008/899,672 cases/controls). This was conducted with both DBP (*n* = 757,601) and SBP (*n* = 757,601). Only DBP results are presented, except for cases in which no DBP analysis could be conducted (ADRA2A and AGTR1). CAD (*n* = 122,733/424,528 cases/controls), stroke (*n* = 67,162/454,450) and heart failure (heart fail., *n* = 14,262/471,898 cases/controls) were used as a positive control and are indicated in red. Targets where the positive control is in the unexpected direction or with no control with a *P* value < 0.1 have been excluded. Data are presented as the causal MR estimate as the center and error bars indicating a ±95% CI, with the minima corresponding to the lower 95% CI bound and the maxima representing the upper 95% CI bound. The number of IVs used in each analysis is given: for targets with multiple IVs, IVW MR was used; for those with a single IV, the Wald ratio was calculated. The *F*-statistic is also provided as a measure of instrument strength. *P* values are uncorrected; multiple-comparison corrected *P* values (applied at differing levels) are available in the Supplementary Information.

**Extended Data Fig. 2:**
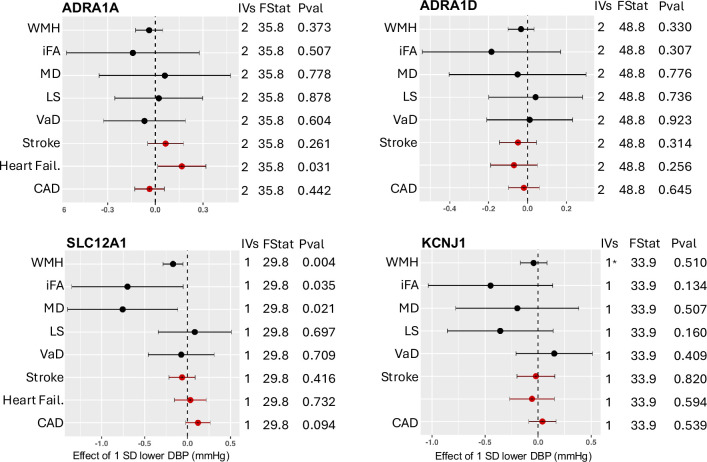
Additional Plots for Antihypertensive Drug Target MR using DBP Downstream Biomarker Data, for which there was no reductive effect found on the positive control. Additional plots for inverse variance-weighted MR (Mendelian randomization) for antihypertensive drug targets using large-scale European genome-wide association study (GWAS) summary statistics. The cis-acting gene region was selected for each target from a DBP (a downstream biomarker) GWAS (n = 757601) as the exposure with five vascular dementia outcomes; WMH (white matter hyperintensities, n = 50559), iFA (inversed fractional anisotropy, n = 31125), MD (mean diffusivity, n = 31147), LS (lacunar stroke, n = 6030/248929 cases/controls), VaD (vascular dementia diagnosis, n = 7008/899672 cases/controls). CAD (coronary artery disease, n = 122733/424528 cases/controls), stroke (n = 67162/454450), and heart failure (Heart Fail., n = 14262/471898 cases/controls) were used as a positive control and are indicated in red; the additional targets presented here did not show the expected lowering effect on the positive control (Pval>0.1). Data are presented as the causal MR estimate as the centre and error bars indicating a +/- a 95% confidence interval (CI), with the minima corresponding to the lower 95% CI bound and the maxima representing the upper 95% CI bound. The number of instrumental variables (IVs) used in each analysis is given, for targets with multiple IVs inverse-variance weighted MR was used, for those with a single IV the Wald ratio was calculated. The F-statistic (Fstat) is also provided as a measure of instrument strength. Pval denotes uncorrected p-values; multiple-comparison corrected p-values (applied at differing levels) are available in the supplement. If the WMH meta-analysis dataset could not be used, the UK-Biobank only WMH dataset is presented instead (indicated by a * next to the IVs). DBP (diastolic blood pressure), SD (standard deviation).

**Extended Data Fig. 3:**
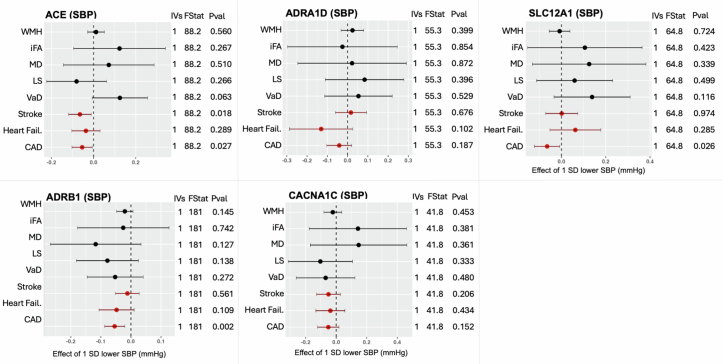
Additional Plots for Antihypertensive Drug Target MR using SBP Downstream Biomarker Data. Additional plots for inverse variance-weighted MR (Mendelian randomization) for antihypertensive drug targets using large-scale European genome-wide association study (GWAS) summary statistics. The cis-acting gene region was selected for each target from a SBP (a downstream biomarker) GWAS (n = 757601) as the exposure with five vascular dementia outcomes; WMH (white matter hyperintensities, n = 50559), iFA (inversed fractional anisotropy, n = 31125), MD (mean diffusivity, n = 31147), LS (lacunar stroke, n = 6030/248929 cases/controls), VaD (vascular dementia diagnosis, n = 7008/899672 cases/controls). CAD (coronary artery disease, n = 122733/424528 cases/controls), stroke (n = 67162/454450), and heart failure (Heart Fail., n = 14262/471898 cases/controls) were used as a positive control and are indicated in red. Data are presented as the causal MR estimate as the centre and error bars indicating a +/- a 95% confidence interval (CI), with the minima corresponding to the lower 95% CI bound and the maxima representing the upper 95% CI bound. The number of instrumental variables (IVs) used in each analysis is given, for targets with multiple IVs inverse-variance weighted MR was used, for those with a single IV the Wald ratio was calculated. The F-statistic (Fstat) is also provided as a measure of instrument strength. Pval denotes uncorrected p-values; multiple-comparison corrected p-values (applied at differing levels) are available in the supplement. SBP (systolic blood pressure), SD (standard deviation).

Figure 3 shows the results of antihypertensive targets for which there was evidence of an effect in the expected direction for at least one of the positive controls: stroke, heart failure and CAD. Results for all other targets with little evidence of an effect on the positive controls are presented in Extended Data Fig. 2 (weighted by DBP) and Extended Data Fig. 3 (weighted by SBP). Results were broadly consistent when using DBP or SBP as the downstream biomarker. There was consistent evidence to support a protective effect of beta-1 adrenergic receptor (ADRB1) antagonists on lower WMH (*β* = −0.03, CI = −0.07–0.001), lower MD (*β* = −0.18, CI = −0.37–0.004), lower VaD risk (OR = 0.90, CI = 0.80–1.01) and lower LS risk (OR = 0.91, CI = 0.80–1.03). There was also evidence of a protective effect of renin on iFA (*β* = −0.53, CI = −0.89 to −0.18) and MD (*β* = −0.42, CI = −0.77 to −0.07) but no other outcomes. For those targets that could only be run using SBP, LS risk was lower with both AGTR1 (OR = 0.77, CI = 0.59–1.00) and ADRA2A (OR = 0.66, CI = 0.48–0.90) modulation, but these targets were not indicated to affect other outcomes. Unexpectedly, there was evidence to suggest ACE inhibition increased risk of VaD; point estimates for WMH, MD and iFA were also in the risk-increasing direction but lacked precision. The VaD risk-increasing estimates for ACE were highly consistent and were replicated in both the SBP and DBP downstream biomarker MR, as well as both protein quantitative trait locus (pQTL) sensitivity analysis MRs (in both deCODE and the UK Biobank Pharma Proteomics Project (UKB-PPP)).

### Anti-inflammatory targets

**Fig. 4:**
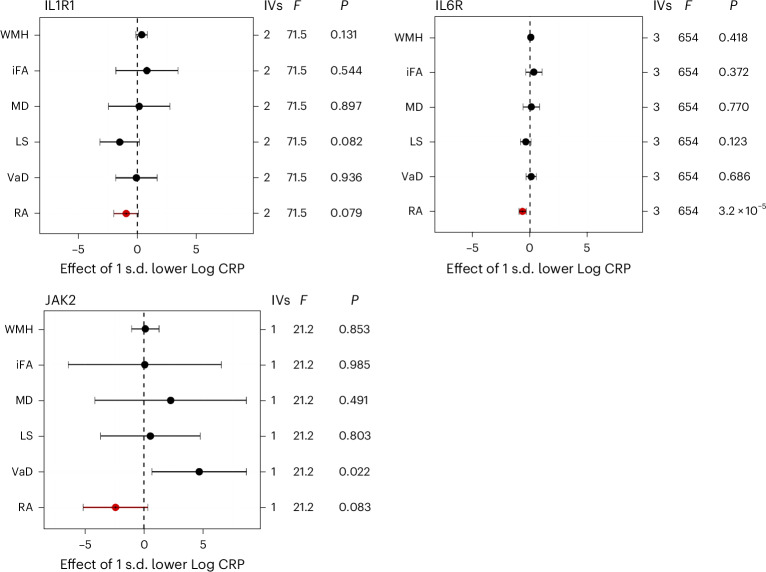
Drug target MR for anti-inflammatory drug targets. IVW MR was conducted for anti-inflammatory drug targets using large-scale European GWAS summary statistics. The *cis*-acting gene region was selected for each target using a downstream biomarker (CRP) as the exposure (*n* = 575,531) with five VaD outcomes: WMH (*n* = 50,559), iFA (*n* = 31,125), MD (*n* = 31,147), LS (*n* = 6,030/248,929 cases/controls), VaD (VaD diagnosis, *n* = 7,008/899,672 cases/controls). RA was used as a positive control and is indicated in red (*n* = 22,350/74,823 cases/controls). The results for all targets with a positive control in the expected direction and with a *P* value < 0.1 are presented. Data are presented as the causal MR estimate as the center and error bars indicating a ±95% CI, with the minima corresponding to the lower 95% CI bound and the maxima representing the upper 95% CI bound. The number of IVs used in each analysis is given: for targets with multiple IVs, IVW MR was used; for those with a single IV, the Wald ratio was calculated. The *F*-statistic is also provided as a measure of instrument strength. *P* values are uncorrected; multiple-comparison corrected *P* values (applied at differing levels) are available in the Supplementary Information.

**Extended Data Fig. 4:**
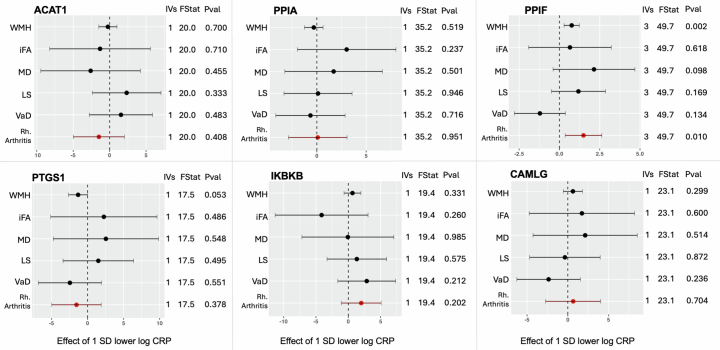
Additional Plots for Anti-inflammatory Drug Target MR using CRP Downstream Biomarker Data, for which there was no reductive effect found on the positive control. Additional plots for inverse variance-weighted MR (Mendelian randomization) for antihypertensive drug targets using large-scale European genome-wide association study (GWAS) summary statistics. The cis-acting gene region was selected for each target from a CRP (a downstream biomarker) GWAS (n = 575531) as the exposure with five vascular dementia outcomes; WMH (white matter hyperintensities, n = 50559), iFA (inversed fractional anisotropy, n = 31125), MD (mean diffusivity, n = 31147), LS (lacunar stroke, n = 6030/248929 cases/controls), VaD (vascular dementia diagnosis, n = 7008/899672 cases/controls).). Rheumatoid arthritis (Rh. arthritis) was used as a positive control and is indicated in red (n = 22350/74823 cases/controls). Data are presented as the causal MR estimate as the centre and error bars indicating a +/- a 95% confidence interval (CI), with the minima corresponding to the lower 95% CI bound and the maxima representing the upper 95% CI bound. The number of instrumental variables (IVs) used in each analysis is given, for targets with multiple IVs inverse-variance weighted MR was used, for those with a single IV the Wald ratio was calculated. The F-statistic (Fstat) is also provided as a measure of instrument strength. Pval denotes uncorrected p-values; multiple-comparison corrected p-values (applied at differing levels) are available in the supplement. The additional targets presented here did not show the expected lowering effect on the positive control (Pval>0.1). CRP (c-reactive protein), SD (standard deviation).

**Extended Data Fig. 5:**
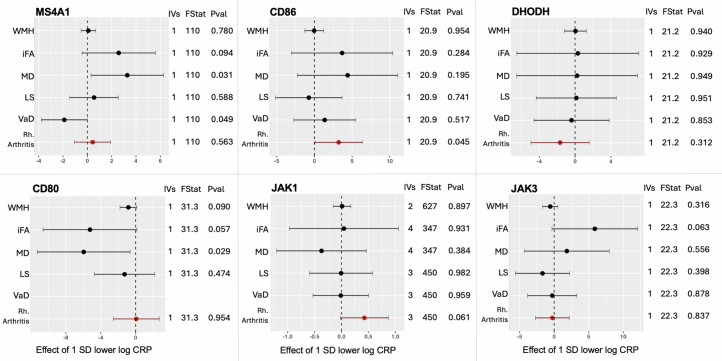
Additional Plots for Anti-inflammatory Drug Target MR using CRP Downstream Biomarker Data, for which there was no reductive effect found on the positive control. Additional plots for inverse variance-weighted MR (Mendelian randomization) for antihypertensive drug targets using large-scale European genome-wide association study (GWAS) summary statistics. The cis-acting gene region was selected for each target from a CRP (a downstream biomarker) GWAS (n = 575531) as the exposure with five vascular dementia outcomes; WMH (white matter hyperintensities, n = 50559), iFA (inversed fractional anisotropy, n = 31125), MD (mean diffusivity, n = 31147), LS (lacunar stroke, n = 6030/248929 cases/controls), VaD (vascular dementia diagnosis, n = 7008/899672 cases/controls).). Rheumatoid arthritis (Rh. arthritis) was used as a positive control and is indicated in red (n = 22350/74823 cases/controls). Data are presented as the causal MR estimate as the centre and error bars indicating a +/- a 95% confidence interval (CI), with the minima corresponding to the lower 95% CI bound and the maxima representing the upper 95% CI bound. The number of instrumental variables (IVs) used in each analysis is given, for targets with multiple IVs inverse-variance weighted MR was used, for those with a single IV the Wald ratio was calculated. The F-statistic (Fstat) is also provided as a measure of instrument strength. Pval denotes uncorrected p-values; multiple-comparison corrected p-values (applied at differing levels) are available in the supplement. The additional targets presented here did not show the expected lowering effect on the positive control (Pval>0.1). TCRP (c-reactive protein), SD (standard deviation).

**Extended Data Fig. 6:**
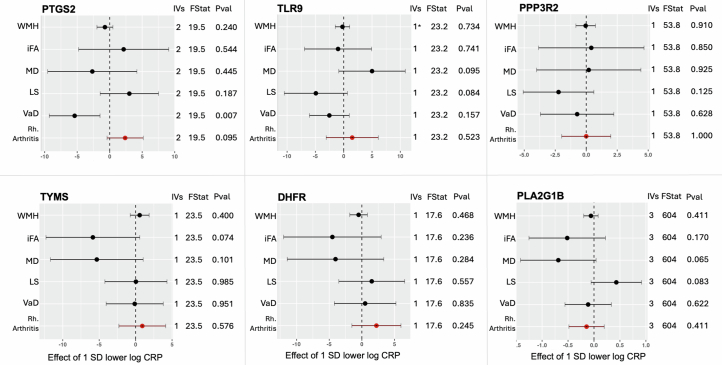
Additional Plots for Anti-inflammatory Drug Target MR using CRP Downstream Biomarker Data, for which there was no reductive effect found on the positive control. Additional plots for inverse variance-weighted MR (Mendelian randomization) for antihypertensive drug targets using large-scale European genome-wide association study (GWAS) summary statistics. The cis-acting gene region was selected for each target from a CRP (a downstream biomarker) GWAS (n = 575531) as the exposure with five vascular dementia outcomes; WMH (white matter hyperintensities, n = 50559), iFA (inversed fractional anisotropy, n = 31125), MD (mean diffusivity, n = 31147), LS (lacunar stroke, n = 6030/248929 cases/controls), VaD (vascular dementia diagnosis, n = 7008/899672 cases/controls).). Rheumatoid arthritis (Rh. arthritis) was used as a positive control and is indicated in red (n = 22350/74823 cases/controls). Data are presented as the causal MR estimate as the centre and error bars indicating a +/- a 95% confidence interval (CI), with the minima corresponding to the lower 95% CI bound and the maxima representing the upper 95% CI bound. The number of instrumental variables (IVs) used in each analysis is given, for targets with multiple IVs inverse-variance weighted MR was used, for those with a single IV the Wald ratio was calculated. The F-statistic (Fstat) is also provided as a measure of instrument strength. Pval denotes uncorrected p-values; multiple-comparison corrected p-values (applied at differing levels) are available in the supplement. The additional targets presented here did not show the expected lowering effect on the positive control (Pval>0.1). If the WMH meta-analysis dataset could not be used, the UK-Biobank only WMH dataset is presented instead (indicated by a * next to the IVs). CRP (c-reactive protein), SD (standard deviation).

Figure 4 shows the results of anti-inflammatory targets for which there was evidence of an effect in the expected direction for the positive control outcome, rheumatoid arthritis (RA). Results for all other targets with little evidence of an effect on this primary positive control outcome are presented in Extended Data Figs. 4–6. Of the 21 anti-inflammatory targets with suitable instruments identified in the downstream biomarker (CRP) GWAS, only three targets showed evidence of an expected effect on RA despite all targets being approved for the treatment of RA. Overall, there was little consistent evidence to suggest a causal effect of any of the anti-inflammatory drug targets on VaD endpoints. Similarly, there was little evidence to suggest causal effects of the targets in the pQTL analysis, and where suggestive evidence was found, it was not consistent across cerebrovascular outcomes or across MR methods. There was suggestive evidence that inhibition of IL1R1 (downstream biomarker analysis) and IL6R (pQTL analysis) were associated with reduced LS risk, but confidence intervals crossed the null. A reduction in JAK2 was associated with increased risk of VaD (downstream biomarker analysis), but there was no evidence of an effect on any other outcomes.

### Sensitivity analyses

**Extended Data Fig. 7:**
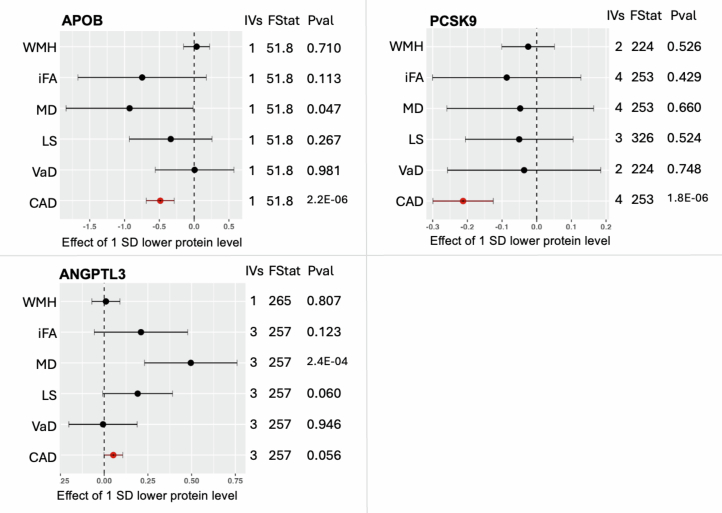
Plots for Lipid-Lowering Drug Target MR using cis-pQTL Data. Inverse variance-weighted MR (Mendelian randomization) was conducted for the lipid-lowering drug target Mendelian randomization using cis-acting pQTLs (n = 35559) as the exposure with five vascular dementia outcomes; WMH (white matter hyperintensities, n = 50559), iFA (inversed fractional anisotropy, n = 31125), MD (mean diffusivity, n = 31147), LS (lacunar stroke, n = 6030/248929 cases/controls), VaD (vascular dementia diagnosis, n = 7008/899672 cases/controls. CAD (coronary artery disease) was used as a positive control and is indicated in red (n = 122733/424528 cases/controls). Data are presented as the causal MR estimate as the centre and error bars indicating a +/- a 95% confidence interval (CI), with the minima corresponding to the lower 95% CI bound and the maxima representing the upper 95% CI bound. The number of instrumental variables (IVs) used in each analysis is given, for targets with multiple IVs inverse-variance weighted MR was used, for those with a single IV the Wald ratio was calculated. The F-statistic (Fstat) is also provided as a measure of instrument strength. Pval denotes uncorrected p-values; multiple-comparison corrected p-values (applied at differing levels) are available in the supplement. SD (standard deviation), pQTL (protein quantitative trait loci).

**Extended Data Fig. 8:**
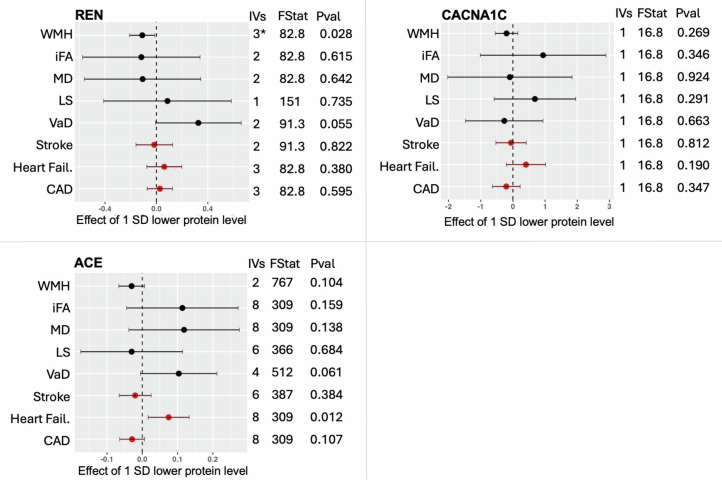
Plots for Anti-hypertensive Drug Target MR using cis-pQTL Data. Inverse variance-weighted MR (Mendelian randomization) was conducted for anti-hypertensive drug target Mendelian randomization using cis-acting pQTLs (n = 35559) as the exposure with five vascular dementia outcomes; WMH (white matter hyperintensities, n = 50559), iFA (inversed fractional anisotropy, n = 31125), MD (mean diffusivity, n = 31147), LS (lacunar stroke, n = 6030/248929 cases/controls), VaD (vascular dementia diagnosis, n = 7008/899672 cases/controls). CAD (coronary artery disease, n = 122733/424528 cases/controls), stroke (n = 67162/454450), and heart failure (Heart Fail., n = 14262/471898 cases/controls) were used as a positive control and are indicated in red. Data are presented as the causal MR estimate as the centre and error bars indicating a +/- a 95% confidence interval (CI), with the minima corresponding to the lower 95% CI bound and the maxima representing the upper 95% CI bound. The number of instrumental variables (IVs) used in each analysis is given, for targets with multiple IVs inverse-variance weighted MR was used, for those with a single IV the Wald ratio was calculated. The F-statistic (Fstat) is also provided as a measure of instrument strength. Pval denotes uncorrected p-values; multiple-comparison corrected p-values (applied at differing levels) are available in the supplement. If the WMH meta-analysis dataset could not be used, the UK-Biobank only WMH dataset is presented instead (indicated by a * next to the IVs). SD (standard deviation), pQTL (protein quantitative trait loci).

**Extended Data Fig. 9:**
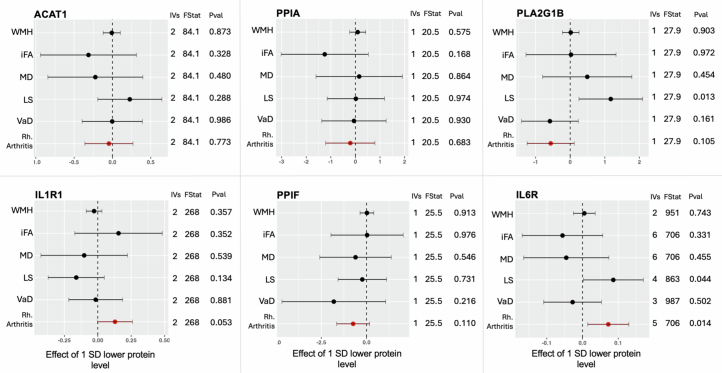
Plots for Anti-inflammatory Drug Target MR using cis-pQTL Data. Inverse variance-weighted MR (Mendelian randomization) was conducted for anti-inflammator drug target Mendelian randomization using cis-acting pQTLs (n = 35559) as the exposure with five vascular dementia outcomes; WMH (white matter hyperintensities, n = 50559), iFA (inversed fractional anisotropy, n = 31125), MD (mean diffusivity, n = 31147), LS (lacunar stroke, n = 6030/248929 cases/controls), VaD (vascular dementia diagnosis, n = 7008/899672 cases/controls).). Rheumatoid arthritis (Rh. arthritis) was used as a positive control and is indicated in red (n = 22350/74823 cases/controls). Data are presented as the causal MR estimate as the centre and error bars indicating a +/- a 95% confidence interval (CI), with the minima corresponding to the lower 95% CI bound and the maxima representing the upper 95% CI bound. The number of instrumental variables (IVs) used in each analysis is given, for targets with multiple IVs inverse-variance weighted MR was used, for those with a single IV the Wald ratio was calculated. The F-statistic (Fstat) is also provided as a measure of instrument strength. Pval denotes uncorrected p-values; multiple-comparison corrected p-values (applied at differing levels) are available in the supplement. SD (standard deviation), pQTL (protein quantitative trait loci).

**Extended Data Fig. 10:**
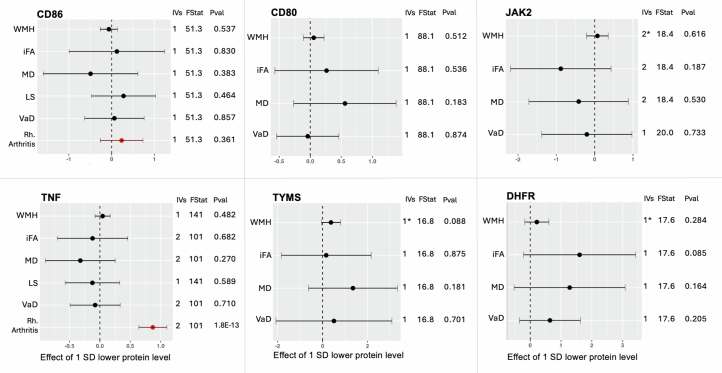
Plots for Anti-inflammatory Drug Target MR using cis-pQTL Data. Inverse variance-weighted MR (Mendelian randomization) was conducted for anti-inflammatory drug target Mendelian randomization using cis-acting pQTLs (n = 35559) as the exposure with five vascular dementia outcomes; WMH (white matter hyperintensities, n = 50559), iFA (inversed fractional anisotropy, n = 31125), MD (mean diffusivity, n = 31147), LS (lacunar stroke, n = 6030/248929 cases/controls), VaD (vascular dementia diagnosis, n = 7008/899672 cases/controls).). Rheumatoid arthritis (Rh. arthritis) was used as a positive control and is indicated in red (n = 22350/74823 cases/controls). Data are presented as the causal MR estimate as the centre and error bars indicating a +/- a 95% confidence interval (CI), with the minima corresponding to the lower 95% CI bound and the maxima representing the upper 95% CI bound. The number of instrumental variables (IVs) used in each analysis is given, for targets with multiple IVs inverse-variance weighted MR was used, for those with a single IV the Wald ratio was calculated. The F-statistic (Fstat) is also provided as a measure of instrument strength. Pval denotes uncorrected p-values; multiple-comparison corrected p-values (applied at differing levels) are available in the supplement. SD (standard deviation), pQTL (protein quantitative trait loci).

Complete results of all sensitivity analyses are in Supplementary Tables 1–15). Extended Data Figs. 7–10 illustrate the *cis*-pQTL MR results for lipid-lowering, antihypertensive and anti-inflammatory targets, and Supplementary Figs. 1–3 show results for the *cis* and *trans* pQTL MR analysis. Overall, the MR sensitivity analyses produced broadly comparable results, with none materially altering our conclusions. In summary, the pQTL sensitivity analyses were largely null. Where associations that were identified in the downstream biomarker analysis could be replicated using pQTLs, we found that the effect of ACE on VaD was consistent across both MR methods. In contrast, no effect of renin on iFA or MD was observed in the pQTL analysis (compared to renin inhibition showing a reductive effect in the downstream biomarker analysis). Although only a limited number of targets had sufficient instruments to allow both generalized inverse-variance weighted (IVW) and cisMR-cML analyses, we found consistent evidence that ADRB1 inhibition is associated with reduced MD and WMH. Colocalization analysis identified little evidence of shared causal variants, with no values with a posterior probability of hypothesis 4 > 50% for targets considered as having a potential causal effect in the MR analysis (positive control in the correct direction and an effect on at least one VaD outcome). Due to using only *cis*-variants, MR–Egger and weighted-median sensitivity analyses could be performed for only five of the 38 targets tested. This increased to 15 targets in the reduced *P*-value threshold (*P* < 5 × 10^−5^) analyses. In both analyses, results remained largely null, and there was little evidence of pleiotropy (Supplementary Tables 2–4 and 7). Given the largely null findings across the 38 targets observed in the main analysis, multiple-testing correction made very little difference to our results (Supplementary Tables 2 and 3).

## Discussion

Overall, our results suggest limited opportunities for repurposing many existing lipid-lowering, antihypertensive and anti-inflammatory drugs for treating VaD. These findings are important as they help guide the prioritization or deprioritization of repurposing candidates, reducing costly investment in options with limited biological plausibility; indeed, evidence has previously shown that drug targets with human genetic evidence are more than twice as likely to achieve regulatory approval^11^. We found consistent evidence for a protective effect of ADRB1 antagonists in reducing VaD risk, LS risk, WMH volume and MD. In addition, genetically indexed ACE inhibition was, unexpectedly, associated with worse cerebrovascular outcomes. There are also some drug classes, such as HMGCR inhibition, for which our results were ambiguous and that clearly warrant further follow-up in relation to cerebrovascular diseases.

**Fig. 5:**
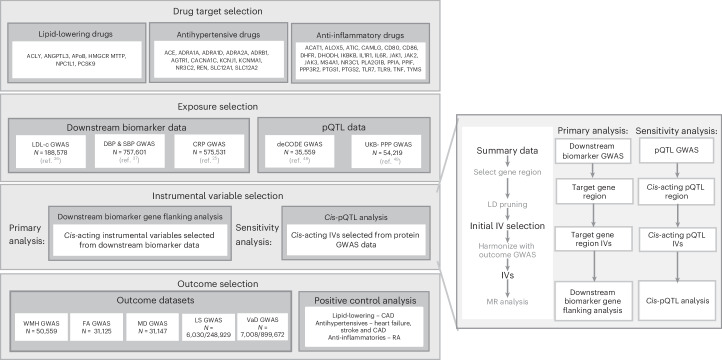
Illustration of data, IV selection and analyses performed. A flowchart summarizing the methods used in this analysis including drug target selection, exposure and outcome selection and obtaining IVs. LD, linkage disequilibrium.

ADRB1 is a G protein-coupled receptor that binds to epinephrine and norepinephrine, leading to physiological effects such as increased heart rate, contractility and cardiac output^17^. ADRB1 is expressed in several brain regions, including the prefrontal cortex and the hippocampus, which play an important role in memory and learning^18^. Although there is a well-established relationship between hypertension and VaD^15^, there remains limited evidence specifically linking beta-blockers to neurological diseases. One study found that patients with hypertension taking beta-blockers capable of crossing the blood-brain barrier had a lower incidence of Alzheimer’s disease compared with those on less permeable beta-blockers^19^, and another found that patients with hypertension who were treated with beta-blockers had better cognitive performance compared with those treated with other antihypertensive medications^20^. An IV analysis using prescribers’ preference as the instrument also reported beta-blockers conferred small protective effects on dementia risk (13 fewer cases (95% CI = 6, 20) of any dementia per 1,000 treated, compared with other antihypertensives). However, none of these studies examined the effects on VaD specifically. Although we found evidence of an effect of ADRB1 antagonism across multiple cerebrovascular disease outcomes and across multiple MR methods that have different underlying assumptions, it was not possible to replicate this effect using ADRB1 pQTLs due to the lack of pQTL data currently available (ADRB1 is not available in UKB, deCODE or other large pQTL resources such as Atherosclerosis Risk in Communities study). When such data become available, future studies should seek to replicate this finding in a pQTL framework. Given the lack of evidence for other blood-pressure-modulating targets, it is plausible that any protective effects of ARDB1 antagonists on VaD risk act through mechanisms independent of blood pressure modulation. This could, in theory, be examined using multivariable MR including both ADRB1 and blood pressure as exposures. However, given that multivariable MR requires conditionally independent IVs for both exposure traits—that is, both ADRB1 and blood pressure—the current lack of pQTL data available for ADRB1 limits the feasibility of performing this analysis. Our finding that ACE inhibition was linked to higher VAD risk seems counterintuitive, particularly given (1) that we observed a protective effect on all-stroke risk and (2) the strong association of blood pressure with increased VaD risk, and the effectiveness of ACE inhibitors for reducing blood pressure. ACE inhibitors lower blood pressure by preventing the production of angiotensin II, a hormone that narrows blood vessels and increases blood volume^21^. Similar adverse effects of ACE inhibitors have been observed previously for dementia risk, both in an observational study with all-cause dementia as an outcome^22^ and in two MR studies with Alzheimer’s disease as an outcome^23,24^. Combined, these findings suggest that ACE inhibition may potentially increase dementia risk: possibly via mechanisms not involving blood pressure modulation, given that few effects were observed for other blood pressure modulating targets. It is also worth noting that AGTR1, another target that also inhibits angiotensin II (by binding to and blocking angiotensin II receptors, rather than inhibiting angiotensin II production like ACE), did not show evidence of an effect on VaD risk. Our study provides a comprehensive analysis of lipid-lowering, antihypertensive and anti-inflammatory drug targets for preventing or treating VaD. We used the largest GWAS available (Table 2) and conducted a replication pQTL analysis, triangulating findings across clinical diagnoses and neuroimaging endophenotypes (see Fig. 5). A key strength of our study is the use of two-sample MR, which reduces confounding by indication and reverse causation inherent in observational pharmacoepidemiology studies of medication use and VaD. Unlike one-sample MR, which requires all data in a single cohort and is therefore often underpowered for rarer outcomes, two-sample MR leverages large GWAS meta-analyses, providing greater statistical power and more reliable inference in this setting. This increases confidence that the effects we observe are causal and may even refute existing observational associations for targets where genetic evidence is null. Given the high cost of clinical trials, particularly in dementia, where long follow-up is required, target prioritization is essential.

**Table 2:** A summary of the sources of the main dataset in the analysis

GWAS		Population	*N*	Source	
**Exposure**					
Downstream biomarkers					
LDL		EUR	1,88,578	Willer et al., 2013	^36^
SBP		EUR	7,57,601	Evangelou et al., 2018	^37^
DBP		EUR	7,57,601	Evangelou et al., 2018	^37^
CRP		EUR	5,75,531	Said et al., 2022	^25^
pQTL data					
deCODE pQTLs	(4,907 proteins)	EUR	35,559	Ferkingstad et al., 2021	^48^
UKB-PPP pQTLs	(2,923 proteins)	EUR	54,219	Sun et al., 2023	^49^
**Outcome**					
VaD					
Meta-analysis		EUR	7,008 / 899,672	Supplementary Information	
FinnGen		EUR	3,116 / 433,066	Kurki et al., 2023	^31^
MEGAVCID		EUR	3,892 / 466,606	MEGAVCID et al., 2024	^30^
Neuroimaging features					
WMH		EUR	50,559	Sargurupremraj et al., 2020	^34^
iFA		EUR	31,125	Taylor-Bateman et al., 2022	^15^
MD		EUR	31,147	Taylor-Bateman et al., 2022	^15^
LS		EUR	6,030 / 248,929	Traylor et al., 2021	^35^
**Positive controls**					
CAD		EUR	122,733 / 424,528	van der Harst et al., 2018	^38^
Stroke		EUR	67,162 / 454,450	Malik et al., 2018	^40^
Heart failure		EUR	14,262 / 471,898	Sakaue et al., 2021	^39^
RA		EUR	22,350 / 74,823	Ishigake et al., 2022	^41^

A list of the main datasets used in this analysis. All are taken from publicly available summary statistics, with the source provided. The number and ethnicity of participants are provided. Full details of each study can be found in the original publications, including the average age and proportion of each sex. Studies were chosen based on the largest available. EUR, European.

**Fig. 6:**
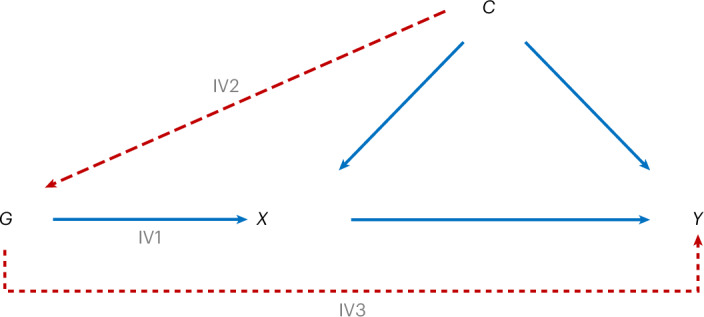
Illustration of MR assumptions. Summary of IV assumptions in MR. *G* represents genetic variants selected as IVs. *X* represents the exposure and *Y* the outcome. *C* represents potential confounders. Each of the three IV assumptions is given on the diagram. IV1: the genetic instrument is strongly associated with the exposure. Red dashed lines indicate that there should be no violations of IV2: there is no confounding of the instrument–outcome relationship. And (IV3) the instruments affect the outcome only via the exposure.

The validity of our analyses relies on satisfying the core assumptions of MR (Fig. 6): (IV1) relevance: the instrument is strongly associated with the exposure; (IV2) independence: no instrument–outcome confounding (for example, by population stratification); and (IV3) exclusion restriction: the instrument must affect the outcome only through the exposure. To address IV1, we selected the strongest available instruments and calculated *F*-statistics to identify and remove weak instruments. For IV2, genetic variants are randomly allocated at conception, making it very unlikely they are affected by lifestyle and sociodemographic traits. We also restricted analyses to individuals of European ancestry, and GWAS summary statistics included in our MR analyses were further adjusted for genetic principal components, minimizing bias due to population stratification. For IV3, to reduce the risk of horizontal pleiotropy, we primarily used *cis*-acting variants with strong biological plausibility. In addition, where possible, we applied MR–Egger to examine the potential for any bias due to pleiotropy, and very little evidence was found. Nonetheless, any violations of these assumptions could bias our effect estimates (for example, residual population stratification or pleiotropy), and thus they should be interpreted as causal only if the underlying assumptions hold.

There are several additional limitations to our study. First, choosing a suitable downstream biomarker for certain drug targets can be challenging. For example, it is unclear that CRP is the most appropriate biomarker for anti-inflammatory drugs (we chose CRP due to its use as a marker of systemic inflammation^25^). In the alternative biomarker sensitivity analyses, we considered interleukin-6 (IL-6); however, only two targets had genetic instruments. White blood cell count was also used as an alternative biomarker to further investigate inflammatory effects. Second, the performance of positive controls was inconsistent across antihypertensive and anti-inflammatory targets, and many targets were discounted due to positive controls not performing well. It is challenging to unpick whether this is because the genetic variants were not relevant and there was a lack of statistical power (a violation of MR assumption of IV1: relevance) or because we did not select the most appropriate positive control for each target. Anti-inflammatory drugs are used to treat many conditions, so a tailored approach to each target is required. RA was the only disease for which all targets were approved to treat in our study; only a small number of targets were approved for treating Crohn’s disease and ulcerative colitis. In other cases, the relationship between specific drug targets and the positive controls may be complex. For example, loop diuretics are prescribed to treat complex hypertension. Still, previous research has shown that in some cases, they may worsen CAD risk^26^. Third, for some targets, there is sample overlap for some analyses. However, the MRlap sensitivity analysis correcting for bias due to sample overlap was broadly consistent. Fourth, we cannot rule out horizontal pleiotropy as an explanation for our findings, as pleiotropy-robust methods could only be applied to five of the 38 tested targets. Nevertheless, where these methods were applicable, there was very little evidence of bias, and given that the majority of associations were null, any residual pleiotropy is unlikely to materially affect our overall conclusions. Fifth, we did not look at any drug–drug interactions, and it is plausible that effects on VaD outcomes may differ if a combination of drug classes are simultaneously targeted (for example, by taking an antihypertensive and a statin). Sixth, our estimates are only relevant to specific target modulation and do not incorporate off-target effects of specific therapeutic agents. Seventh, although the largest datasets available were chosen, sample sizes were still relatively small, particularly for clinically diagnosed VaD, potentially limiting statistical power. Eighth, all neuroimaging biomarker GWASs primarily consisted of UKB participants; thus, average ages of the samples were relatively young for dementia pathology. We may have observed different findings using a GWAS of an older population, but as yet these do not exist. Ninth, we cannot rule out false positives due to multiple-testing bias; however, results remained largely similar after applying three complementary multiple-testing corrections. Instead, an approach of replication using additional datasets (for example, pQTLs) and identifying consistent patterns of effects across multiple related outcomes was used to determine key findings. Finally, all analyses were restricted to European ancestry samples due to data availability, and results may not generalize to other ancestry groups.

ADRB1 antagonists appear to be a promising candidate for potential repurposing; future studies should examine the mechanisms through which ADRB1 modulates VaD risk. ACE inhibitors may increase vascular (and other) dementia risk, and pharmacovigilance studies are required to confirm this effect. There was little evidence that other lipid-lowering, antihypertensive or anti-inflammatory drugs could be repurposed to prevent or treat VaD.

## Methods

### Drug target selection

Figure 5 provides an overview of the data and methods used in our study. For each class of medication considered (lipid-lowering, antihypertensive and anti-inflammatory), the key drug targets of licensed therapeutics were identified using a combination of the British National Formulary (https://bnf.nice.org.uk/) and National Institute for Health and Care Excellence (www.nice.org.uk) guidelines. Our search resulted in 46 potential targets (Table 1). We identified each drug’s target using https://go.drugbank.com/ (ref. ^27^) and determined the coordinates of protein-coding gene region(s) using https://www.ncbi.nlm.nih.gov/gene (ref. ^28^).

### Data

#### Outcome data

We considered five different VaD outcomes: VaD clinical diagnosis, WMH volume, iFA, MD and LS. For VaD clinical diagnosis, we conducted a meta-analysis of two existing case-control GWAS using METAL (full details in the Supplementary Information)^29^, combining summary data from the Mega Vascular Cognitive Impairment and Dementia consortium (MEGAVCID)^30^ (*N* cases = 3,892, *N* controls = 466,606) and the FinnGen study^31^ (*N* cases = 3,116, *N* controls = 433,066). This gave a maximum of *N* = 7,009 cases and *N* = 899,672 controls. We also considered four neuroimaging features of VaD: WMH and LS are used in clinic for diagnosis and prognosis^32^, and Diffusion Tensor Imaging measures (MD and FA) are primarily used in research and prognostic studies and are considered promising biomarkers of microstructural white matter damage^33^. For ease of interpretation, the directionality of FA was inversed (called iFA throughout) to match the direction of the other VaD outcomes. Thus, higher values of all traits represent worse cerebrovascular health. Summary-level GWAS data for these traits were obtained from the largest, most recent studies: WMH (*N* = 50,559)^34^; FA and MD (*N* = 31,125 and 31,147, respectively)^15^; and LS (*N* = 7,338 cases, 254,798 controls)^35^. A smaller WMH GWAS dataset (*N* = 31,855)^15^ was used if variants that met the genome-wide threshold for drug targets were not available in the primary WMH meta-analysis.

### Downstream biomarker data

GWAS summary data information can be found in Table 2. GWAS summary data were obtained for four downstream biomarkers (that is, traits expected to be targeted by each drug). These included LDL-c for lipid-lowering targets (ref. ^36^, *n* = 188,578); SBP and DBP (ref. ^37^, *n* = 757,601) for antihypertensive targets and CRP for anti-inflammatory targets (ref. ^25^, *n* = 575,531). The interpretation of results is per unit increase in the downstream biomarker (that is, per 1 mmHg increase for SBP and DBP, per s.d. increase for LDL-c and per 1 unit increase in natural log-transformed CRP). For example, using SBP as the downstream biomarker, a MR odds ratio of 0.90 reflects the effect per 1 mmHg lower SBP as instrumented via genetic variation in the target locus. A list of chosen IVs can be found in Supplementary Table 16. An initial MR analysis of the downstream biomarkers on VaD risk was also conducted to evaluate their potential utility (a full description of the methods can be found in the Supplementary Methods).

#### Positive control data

We evaluated instrument validity using positive control traits (that is, disorders each drug is licensed to treat) as additional outcomes. For lipid-lowering drug targets, we considered CAD (*N* cases = 122,733, *N* controls = 424,528)^38^; for antihypertensive targets, we considered heart failure (*N* cases = 22,350, *N* controls = 74,823)^39^, stroke (*N* cases = 67,162, *N* controls = 454,450)^40^ and CAD^38^; for anti-inflammatory targets, we considered RA (*N* cases = 22,350, *N* controls = 74,823)^41^.

### Statistics and reproducibility

#### Statistical analysis

All analyses were conducted using R (v.4.4.0). Two-sample MR was the primary analysis method, which uses existing GWAS summary data for both the exposure and the outcome (Fig. 6). MR relies on three key underlying assumptions: (1) the genetic instrument is strongly associated with the exposure, (2) there is no confounding of the instrument–outcome relationship and (3) the instruments affect the outcome only via the exposure. Violation of any of these assumptions can bias the validity of the causal effect estimates. Further details on the principles and application of MR can be found in ref. ^42^ and the Supplementary Methods. No statistical method was used to predetermine sample size; instead, the largest publicly available summary datasets that avoided sample overlap (where possible) were used. A list of cohorts used in each of the summary statistics used in this study as provided in the original source publications can be found in Supplementary Table 17. Detailed information on participants used in those studies can be found in the original papers, including average age and sex.

For each drug target, *cis*-acting genetic variants (within a 500-kb region on either side of the target gene), which were associated with the downstream biomarker at genome-wide threshold (*P* < 5 × 10^−8^), were identified^43^. Independent instruments were selected using a linkage disequilibrium clumping threshold of *r*^2^ < 0.001 within a 10,000-kb distance. The *P*-value threshold was lowered if no instruments were identified at genome-wide threshold to *P* < 5 × 10^−5^. It is worth noting that there are limitations to lowering the *P*-value threshold, as it increases the risk of using potentially invalid instruments and horizontal pleiotropy, which may bias our results. All effect alleles were aligned to harmonize data across the drug target and VaD outcome datasets^43^. The number of IVs used for each analysis and the corresponding *F*-statistics, which measure instrument strength, were calculated. Targets with an *F-*statistic below 10 were interpreted to be susceptible to weak instrument bias. Two-sample MR was performed using a Wald-ratio approach (if only one genetic instrument was available) or an IVW (if more than one genetic instrument was available) as the primary analysis with the ‘MendelianRandomization’ R package^44,45^. Full details of the Wald-ratio and IVW methods are in the Supplementary Information. Where the number of IVs allowed, Cochran’s *Q* statistics and additional MR sensitivity analyses were calculated to examine potential bias due to horizontal pleiotropy. Application of weighted-median MR^46^ and MR–Egger^47^ weighted-median MR was possible only for targets with three more instruments. A relaxed instrument threshold (*P* < 5 × 10^−5^) was applied to increase the number of targets for which we could perform pleiotropy-robust methods, but it is worth noting that reducing the *P-*value threshold increases risk of including invalid (and potentially pleiotropic) instruments. At least one positive control was evaluated as an outcome for each drug class (CAD for lipid-lowering drugs; heart failure, CAD and stroke for antihypertensive drugs; and RA for anti-inflammatory drugs) to validate instrument validity.

#### Sensitivity analyses

Several sensitivity analyses were conducted to assess robustness of the findings. These included (1) replication using pQTLs rather than downstream biomarkers as the exposure (this analysis was conducted in two independent pQTL datasets and via meta-analysis of those two pQTL datasets^48,49^ to increase statistical power); (2) pQTL based *trans*-acting MR analyses to maximize the number of instruments; (3) MRlap^50^, a method that is robust to partial and complete sample overlap to account for UKB being present in multiple exposure and outcome datasets; (4) *cis*-acting generalized IVW MR^51^ and cisMR-cML^52^, to incorporate correlated instruments and thereby increase the number of IVs; (5) statistical colocalization as an alternative approach with different assumptions, applied to each *cis*-acting gene region to examine whether the same causal variant was identified for the exposure and the outcome; (6) use of alternative downstream biomarkers (IL-6, white blood cell count, triglycerides and blood pressure unadjusted for body mass index) to further validate results; (7) alternative positive controls for anti-inflammatory targets (Crohn’s disease and ulcerative colitis) to further validate our instruments; and (8) MR using proxy single nucleotide polymorphisms in place of missing IVs to maximize the number of IVs. Full details of these sensitivity analyses are provided in the Supplementary Methods, and additional GWAS datasets used in the sensitivity analysis can be found in Supplementary Table 18. To account for multiple comparisons across 38 protein targets and five correlated VaD outcomes, we implemented a multistep correction strategy. Outcome-specific MR *P* values were first combined using the Cauchy combination test (ACAT)^53^ and adjusted using the Li and Ji method^54^ to reflect the effective number of independent outcomes (*M*_eff_ = 4). These ACAT + *M*_eff_-adjusted *P* values were then corrected for multiplicity using the false discovery rate (FDR)^55^ both within biological drug classes (that is, lipid-lowering, antihypertensive and anti-inflammatory) and across all targets. These three complementary steps—ACAT + *M*_eff_, within-class FDR and global FDR—account for outcome correlation and hierarchical biological structure. Full details are provided in the Supplementary Methods. All analyses followed Strengthening the Reporting of Observational Studies in Epidemiology using MR guidelines (STROBE-MR)^56^; the corresponding checklist is included in the Supplementary Information.

### Reporting summary

Further information on research design is available in the Nature Portfolio Reporting Summary linked to this article.

## Supplementary information

Supplementary Information
Supplementary Methods, Figs. 1–3, Tables 1–17 and an index with descriptors.

Reporting Summary

Supplementary Tables 1–17

## Data Availability

All summary statistics were obtained from publicly available datasets, with details from the original papers as described in Table 2, refs. ^15,25,30,31,34–41,48,49^, and from the Neale Lab UK Biobank resource (http://www.nealelab.is/uk-biobank/). Further details can also be found in Supplementary Table 1.

## Code availability

Code used in this study is available via Zenodo at 10.5281/zenodo.15190762 (ref. ^57^).
