# Supplementary material for: Repurposing drugs for the prevention of vascular dementia using evidence from drug target Mendelian randomization
Source: Nat Aging. 2026 Apr 20;6(4):905–15. doi: 10.1038/s43587-026-01106-1 (PMC13099373; doi:10.1038/s43587-026-01106-1)
Supplement: Supplementary file 1 — Supplementary Information Supplementary Methods, Figs. 1–3, Tables 1–17 and an index with descriptors. [file 43587_2026_1106_MOESM1_ESM.pdf]

nature aging

Supplementary information https://doi.org/10.1038/s43587-026-01106-1

Repurposing drugs for the prevention of vascular dementia using evidence from drug target Mendelian randomization

In the format provided by the authors and unedited

<u>Supplement Index:</u>

Supplementary Methods 2-8
Additional Data Sources 2
Protein Quantitative Trait Loci 2
Vascular Dementia Meta-Analysis 2
Additional Statistical Analysis 3
Initial Downstream Biomarker Mendelian Randomization 3
MR-Egger and Weighted Median Mendelian Randomization 3
Multiple Testing Correction 4
Additional Positive Controls and Alternative Biomarkers 5
MRlap for Overlapping Samples 5
Mendelian Randomization with Proxy IVs 5
Mendelian Randomization with Correlated IVs 6
pQTL Sensitivity Analysis 6
Statistical Colocalization Analysis 7
Supplementary References 8
Supplementary eFigures 10
eFigure 1: Lipid-Lowering Drug Target MR using *cis* and *trans* pQTL Instruments for Analysis 10
eFigure 2: Anti-hypertensive Drug Target MR using *cis* and *trans* pQTL Instruments for Analysis 11
eFigure 3: Anti-inflammatory Drug Target MR using *cis* and *trans* pQTL Instruments for Analysis 12-14

Supplementary Methods:

Additional Data Sources

Protein Quantitative Trait Loci Data

All analyses were replicated using pQTL data where available. GWAS summary data from the largest scale publicly available European genome-wide association studies (GWAS) were obtained for 46 drug targets (where available) from the deCODE cohort (Ferkingstad *et al*., 2021, n=35,559)[1] and the UK Biobank PPP study (Sun *et al*., 2023, n= 54,219)[2]. Where possible, primary analyses were conducted using deCODE data, as this prevented participant overlap with all selected outcome studies, which were primarily measured in the UK Biobank. Where no deCODE data was available, UKB-PPP data was used instead. The interpretation of results from this analysis is per standard deviation (SD) increase in protein levels.

The deCODE study collected data using the using SomaScan multiplex aptamer assay (version 4). This uses protein-capture SOMAmer (Slow Offrate Modified Aptamer) reagents to bind to specific protein targets for quantification using DNA microarrays[1]. In total 4,907 aptamers representing 4,719 plasma proteins were measured with GWAS summary data available from https://www.decode.com/summarydata/. The UK Biobank Pharma Proteomics Project (UKB-PPP) study measured 2,923 plasma proteins using the Olink Explore 3072 PEA (Proximity Extension Assay). This method utilizes probes formed from antibodies and oligonucleotides generating DNA-encoded tags for measurement[2]. The pQTL data used is publicly available at https://www.synapse.org/Synapse:syn51364943/wiki/622119.

Vascular Dementia Meta-Analysis

A meta-analysis for vascular dementia was performed using the METAL toolkit[3]. This combined summary data from two recently published European GWAS. Firstly, data from MEGAVCID consortium (MEGAVCID *et al*., 2024[4]) which has N cases = 3,892, N controls = 466,606. This study combined data from 11 cohorts with VaD cases (primarily defined using International Statistical Classification of Diseases and Related Health Problems (ICD) codes) plus an additional 4 providing only controls[4]. Secondly, the FinnGen study[5] (N cases = 3,116, N controls = 433,066). The FinnGen study is a large-scale genomics initiative that has analyzed over 500,000 Finnish biobank samples and correlated genetic variation with health data to understand disease mechanisms and predispositions. The project is a collaboration between research organizations and biobanks within Finland and international industry partners. Cases

were identified using the “F5_VASCDEM” endpoint which is based on the digital health record data from Finnish health registries (ICD-10: F01). An inverse-variance weighted meta-analysis was conducted to combine the studies. This gave a maximum of N= 7,008 cases and N= 899,672 controls in the meta-analysis. There was no evidence of genomic inflation in the two source GWAS (lambdas within 0.1 of 1) so no genomic control was applied during the meta-analysis.

Additional Statistical Analysis

All analysis was administered in R (version 4.4.0). Instrumental variable selection and harmonization was conducted using the ‘TwoSampleMR’ and ‘ieugwasr’ packages[6]. MR analysis was performed using the ‘MendelianRandomization’ R package[7, 8]. Additional sensitivity analysis was conducted using the ‘coloc’ and ‘MRlap’ R packages[9, 10, 11]. The authors acknowledge the use of the UCL Myriad High Performance Computing Facility (Myriad@UCL), and associated support services, in the completion of this work.

Initial Downstream Biomarker Mendelian Randomization

To evaluate the overall link between the selected downstream biomarkers and vascular dementia, a Mendelian randomization comparison between SBP, DBP, LDL-c and CRP with VaD risk was conducted. Genome-wide significant instruments were selected at a $5x10^{-8}$ p-value threshold and an LD clumping threshold of $r^2 < 0.001$ within a 10,000kb distance. The number of instruments selected for each target can be found in supplementary table 1. For CRP instruments were restricted to the *CRP* gene region (±500 kb). IVW/Wald ratio, weighted median and MR-Egger methods were all applied.

MR-Egger and Weighted Median Mendelian Randomization

Whilst the inverse-variance weighted (IVW) MR and Wald-ratio methods (used as our primary analysis method) have the best statistical power, it does rely on stringent instrument assumptions[12]. These assume no unbalanced horizontal pleiotropy (i.e., no effect of the instruments on the outcomes through any pathway other than the drug target). Thus, the intercept in these models is fixed at zero. If there is horizontal pleiotropy (i.e. an effect of the instruments on the outcomes that does not go through the drug target, otherwise known as pre-translational pleiotropy), the effect estimate for that specific drug target will be biased. Other MR methods have more relaxed assumptions. In order to address potential violations of these assumptions additional sensitivity analysis was conducted. Weighted Median MR combines the

Wald ratio estimates for each genetic variant with the median inverse-variance weight[13]. Unlike IVW MR, weighted median MR still provides a consistent estimate as long as at least 50% of the weights are from valid instruments. MR-Egger is a MR method designed to estimate the magnitude of and adjust for horizontal pleiotropy, by not constraining the intercept term to zero. Instead, the intercept estimates the average pleiotropic effect[14], and the slope provides a pleiotropy corrected causal effect estimate. Consequently, this method allows instruments to be used that are invalid under the third IVW MR assumption (exclusion restriction) and instead relies on the weaker InSIDE (Instrument Strength Independent of Direct Effect) assumption, that pleiotropic effects are independently distributed from the genetic associations with the risk factor. Both MR egger and weighted median MR were applied in addition to the IVW method (using the already selected cis-acting instruments) with the 'MendelianRandomization' R package[7, 8]. Both methods require 3 instrumental variables for analysis, so in many cases were unable to be performed, unless the p-value threshold was reduced from $p< 5x10^{-8}$. Therefore, a reduced threshold sensitivity analysis was conducted. We re-analysed results using a reduced threshold for instrumental variable selection ($p< 5x10^{-5}$, $r^2 < 0.01$ and a 1,000kb distance threshold) to enable pleiotropy robust MR methods, which require more than two SNPs as instruments.

Multiple Testing Correction

Mendelian randomization (MR) analyses were performed for 38 targets grouped into three pharmacological classes (antihypertensive, lipid-lowering, and antidiabetic) across five correlated outcomes. To account for multiple testing while considering biological and statistical dependencies, a three-level hierarchical correction framework was applied. We calculated the effective number of independent tests (Meff) using the Li & Ji method[15] implemented in the 'poolr' R package[16], which accounts for correlations among outcomes. For the 5 outcomes, Meff was estimated to be 4. Firstly, for each protein, MR p-values across outcomes were combined using the Aggregated Cauchy Association Test (ACAT)[17] and subsequently adjusted for the effective number of independent outcomes (Meff = 4), yielding a single outcome adjusted p-value per target. This accounts for correlation among outcomes while conservatively controlling for the total number of outcomes tested. Drug-class-level significance was then assessed using a within-class false discovery rate (FDR)[18] correction applied all targets within each drug class using the outcome-adjusted p-values. Finally, global FDR correction across all 38 targets was applied using the outcome adjusted p-values to identify individual significant targets. Positive control outcomes and supplementary exposures were excluded from multiple testing correction.

This process was repeated for the pQTL supplementary analysis. These approaches attempt to balance the control of false discoveries with the structure of correlated outcomes and biologically related drug groups. However, as the majority of associations were null the FDR-adjusted p-values were 1 or near 1. Results are presented in supplementary tables 2-3.

Additional Positive Controls and Alternative Biomarkers

Due to low case numbers in the RA GWAS, we examined additional positive controls for anti-inflammatory targets as a sensitivity analysis. These were Crohn's disease (N cases= 12,194, N controls= 28,072)[19] and ulcerative colitis (N cases= 12,366, N controls= 33,609)[19]. To further evaluate whether LDL-c was a suitable downstream biomarker for all lipid-lowering drug targets, we examined results when using triglyceride data instead of LDL-c data from Willer *et al*., 2013 (N= 177,861)[20]. We also tested interleukin-6 (IL-6) (N= 21,758)[21] and white blood cell count (WBC) (N= 563,946)[22] instead of CRP as a downstream biomarker for anti-inflammatory targets. As the blood pressure datasets are adjusted for BMI, an additional analysis was conducted using unadjusted blood pressure measurements from UK-Biobank (SBP N= 317,754 DBP N= 317,756)[23]. These datasets are described in supplementary table 18.

MRlap for Overlapping Samples

Overlapping samples in the exposure and outcome data in two-sample MR can result in bias[11]. In our analysis, several datasets contained data from UK Biobank; of the initial data selected as downstream biomarkers for the exposure, only the LDL-c dataset did not contain any UK Biobank participants. To assess for potential bias caused by sample overlap where applicable, 'MRlap'[11] was applied as a sensitivity analysis. MRlap leverages cross-trait LD-score regression (LDSC) to approximate the overlap between summary datasets, before performing an MR analysis. It is then able to output both the original and overlap corrected results; any difference between these two indicates possible bias introduced through sample overlap. A *cis*-acting analyses was then run for all three downstream biomarkers with UK Biobank participants (SBP, DBP & CRP) with outcomes containing UK Biobank participants (WMH, FA & MD). As this method requires 3 instrumental variables for analysis, a reduced instrument selection threshold was used ($p < 5x10^{-5}$, $r^2 < 0.01$ and a 1,000kb distance threshold) in the *cis*-acting region.

Mendelian Randomization with Proxy IVs

To mitigate the effect of missing instrument data in the main cis-acting downstream biomarker Mendelian randomization analysis we used PLINK to search for proxies for IVs that were

missing outcome data. The following parameters were used to search for suitable proxies a window of 99,999 SNPs and ±500 kb distance and a minimum $r^2 \geq 0.2$. A 1000G European reference dataset[24] was used. Where multiple proxies were found the SNP present in both datasets with the highest r^2 with the original IV was used. The following proxies were identified; rs7513374 (for rs146551445, r^2= 0.296); rs12632423 (for rs73082723, r^2= 0.283); and rs2846695 (for rs66518071, r^2= 0.359). Cis-acting MR analysis was then repeated using the established pipeline with results available in supplementary table 14.

Mendelian Randomization with Correlated IVs

The standard drug-target MR approach uses cis-acting instrumental variables which minimizes the introduction of pleiotropy[25]. This does result in a limited number of IVs being selected. Additional MR methods allowing the selection of correlated instruments were also used to perform an analysis with greater numbers of instruments. The methods were a cis-acting generalized IVW MR analysis[26] (conducted at high ($p < 5x10^{-8}$) and relaxed ($p < 1x10^{-5}$) p-value thresholds) and cisMR-cML[27]. These methods account for correlation (linkage disequilibrium) between variants, allowing the inclusion of multiple, potentially correlated instruments while providing a weighted estimate of the causal effect. Generalized IVW MR was performed following the instructions provided (https://yanglab.westlake.edu.cn/software/gcta/#Overview). A European 1000G reference dataset was used for the analysis[24]. Identification of the cis-acting region was as above in the downstream biomarker data. The following parameters were used for analysis; minimum number of SNPs = 5; LD threshold of $r^2 < 0.05$ at a ±500 kb distance. Analysis was performed at a standard genome-wide significance threshold ($p < 5x10^{-8}$) as well as a relaxed threshold ($p < 1x10^{-5}$) to boost IV numbers. Results can be found in supplementary tables 11 and 12 respectively. The cisMR-cML analysis was performed according to instructions available at (https://github.com/ZhaotongL/cisMRcML). Instrumental variables were selected using GCTA-COJO[26] using the default parameters ($p < 5x10^{-8}$) with the cis-acting region as defined previously. cisMR-cML was then conducted on all targets with at least 3 IVs identified, results can be found in supplementary table 13.

pQTL Sensitivity Analysis

Where data was available, results were replicated using pQTLs from deCODE[1] or the UK Biobank[2]. Cis-acting instruments were selected based on a 500kb distance either side of the target gene region. Instruments were selected based on genome-wide significance and an LD clumping threshold of $r^2 < 0.001$ within a 10,000kb distance. For those pQTLs with no genome-

wide significant instruments a reduced threshold of $5x10^{-5}$ was applied instead. As with the main analysis IVW/Wald ratio, weighted median and MR-Egger was applied for analysis with all five VaD outcomes.

An additional analysis including *trans*-acting instrumental variables was conducted. This can capture potential effect(s) of the drug target not occurring within the immediate gene region and provides results for drug targets where no *cis*-acting instruments were identified. It is worth noting that *trans*-acting instruments have an increased risk of horizontal pleiotropy biasing the analysis. A combined *cis* and *trans*-acting analyses were performed using pQTL summary data. Independent instruments (i.e., variants associated with target protein levels at genome-wide significance and an LD clumping threshold of $r^2 < 0.001$ within a 10,000kb distance) were selected across the whole genome. Instruments were identified for 21 drug targets for MR analysis.

A meta-analysis of pQTL summary data from both deCODE (Ferkingstad *et al*., 2021, n=35,559)[1] and the UK Biobank PPP study (Sun *et al*., 2023, n= 54,219)[2] was conducted for 12 proteins with analysis present in both (ACE, ANGPTL3, APOB, CD80, CD86, IL1R1, IL6R, PCSK9, PLA2G1B, PPIF, REN, TNF). This took the same approach using METAL[3] to conduct a meta-analysis as the vascular dementia meta-analysis discussed previously. Inverse-variance weighted meta-analyses were conducted to combine the studies. This gave a maximum of N=89,778 in the meta-analysis. A cis-acting IVW MR was performed using the meta-analysis data following the same pipeline as the individual pQTL MR analyses. Full results can be found in supplementary table 15.

Statistical Colocalization Analysis

Statistical colocalization analyses were performed to evaluate whether associations between the instrument and the drug target and the instrument and the outcomes were driven by the same causal variants using the “coloc” R package[9, 10]. Colocalization analysis tests each variant-level hypothesis with the rest (support for pairings of causal and non-causal variants) using a Bayes factor. As a Bayesian method, three informative prior probabilities are specified (that any random genetic variant in the given region is associated with trait 1, 2 or both)[9]. The method then calculates the following posterior probabilities; PP.H0: there is no association with either trait in the given region; PP.H1: there is an association with only trait 1; PP.H2: there is an association with only trait 2; PP.H3: there is an association with both traits, but they have

different single causal variants; PP.H4: there is an association with both traits and they share the same single causal variant. A lack of colocalization evidence does not necessarily discount a causal effect found in MR, however it does increase the robustness of the results[28]. This was performed for both a 500kb region around each target gene as well as a 500kb region surround each instrument identified in the combined cis and trans pQTL analysis.

Supplementary References

e1. Ferkingstad E, Sulem P, Atlason BA, et al. Large-scale integration of the plasma proteome with genetics and disease. *Nat Genet*. 2021;53(12):1712-1721. doi:10.1038/s41588-021-00978-w

e2. Sun BB, Chiou J, Traylor M, et al. Plasma proteomic associations with genetics and health in the UK Biobank. *Nature*. 2023;622(7982):329-338. doi:10.1038/s41586-023-06592-6

e3. Willer CJ, Li Y, Abecasis GR. METAL: fast and efficient meta-analysis of genomewide association scans. *Bioinformatics*. 2010;26(17):2190-2191. doi:10.1093/bioinformatics/btq340

e4. The Mega Vascular Cognitive Impairment and Dementia (MEGAVCID) consortium. A genome-wide association meta-analysis of all-cause and vascular dementia. *Alzheimer's & Dementia*. 2024;20(9):5973-5995. doi:10.1002/alz.14115

e5. Kurki MI, Karjalainen J, Palta P, et al. FinnGen provides genetic insights from a well-phenotyped isolated population. *Nature*. 2023;613(7944):508-518. doi:10.1038/s41586-022-05473-8

e6. Hemani G, Zheng J, Elsworth B, et al. The MR-Base platform supports systematic causal inference across the human phenome. Loos R, ed. *eLife*. 2018;7:e34408. doi:10.7554/eLife.34408

e7. Burgess S, Butterworth A, Thompson SG. Mendelian randomization analysis with multiple genetic variants using summarized data. *Genet Epidemiol*. 2013;37(7):658-665. doi:10.1002/gepi.21758

e8. Patel A, Ye T, Xue H, et al. MendelianRandomization v0.9.0: updates to an R package for performing Mendelian randomization analyses using summarized data. *Wellcome Open Res*. 2023;8:449. doi:10.12688/wellcomeopenres.19995.1

e9. Wallace C. Eliciting priors and relaxing the single causal variant assumption in colocalisation analyses. *PLOS Genetics*. 2020;16(4):e1008720. doi:10.1371/journal.pgen.1008720

e10. Giambartolomei C, Vukcevic D, Schadt EE, et al. Bayesian Test for Colocalisation between Pairs of Genetic Association Studies Using Summary Statistics. *PLOS Genetics*. 2014;10(5):e1004383. doi:10.1371/journal.pgen.1004383

e11. Mounier N, Kutalik Z. Bias correction for inverse variance weighting Mendelian randomization. *Genetic Epidemiology*. 2023;47(4):314-331. doi:10.1002/gepi.22522

e12. Sanderson E, Glymour MM, Holmes MV, et al. Mendelian randomization. *Nat Rev Methods Primers*. 2022;2(1):1-21. doi:10.1038/s43586-021-00092-5

e13. Bowden J, Davey Smith G, Haycock PC, Burgess S. Consistent Estimation in Mendelian Randomization with Some Invalid Instruments Using a Weighted Median Estimator. *Genet Epidemiol*. 2016;40(4):304-314. doi:10.1002/gepi.21965

e14. Bowden J, Davey Smith G, Burgess S. Mendelian randomization with invalid instruments: effect estimation and bias detection through Egger regression. *Int J Epidemiol*. 2015;44(2):512-525. doi:10.1093/ije/dyv080

e15. Li J, Ji L. Adjusting multiple testing in multilocus analyses using the eigenvalues of a correlation matrix. *Heredity*. 2005;95(3):221-227. doi:10.1038/sj.hdy.6800717
e16. Cinar O, Viechtbauer W. The poolr Package for Combining Independent and Dependent p Values. *Journal of Statistical Software*. 2022;101:1-42. doi:10.18637/jss.v101.i01
e17. Liu Y, Chen S, Li Z, Morrison AC, Boerwinkle E, Lin X. ACAT: A Fast and Powerful p Value Combination Method for Rare-Variant Analysis in Sequencing Studies. *Am J Hum Genet*. 2019;104(3):410-421. doi:10.1016/j.ajhg.2019.01.002
e18. Benjamini Y, Hochberg Y. Controlling the False Discovery Rate: A Practical and Powerful Approach to Multiple Testing. *Journal of the Royal Statistical Society: Series B (Methodological)*. 1995;57(1):289-300. doi:10.1111/j.2517-6161.1995.tb02031.x
e19. de Lange KM, Moutsianas L, Lee JC, et al. Genome-wide association study implicates immune activation of multiple integrin genes in inflammatory bowel disease. *Nat Genet*. 2017;49(2):256-261. doi:10.1038/ng.3760
e20. Willer CJ, Schmidt EM, Sengupta S, et al. Discovery and refinement of loci associated with lipid levels. *Nat Genet*. 2013;45(11):1274-1283. doi:10.1038/ng.2797
e21. Folkersen L, Gustafsson S, Wang Q, et al. Genomic and drug target evaluation of 90 cardiovascular proteins in 30,931 individuals. *Nat Metab*. 2020;2(10):1135-1148. doi:10.1038/s42255-020-00287-2
e22. Vuckovic D, Bao EL, Akbari P, et al. The Polygenic and Monogenic Basis of Blood Traits and Diseases. *Cell*. 2020;182(5):1214-1231.e11. doi:10.1016/j.cell.2020.08.008
e23. UK Biobank. Neale lab. Accessed March 21, 2025. http://www.nealelab.is/uk-biobank
e24. Auton A, Abecasis GR, Altshuler DM, et al. A global reference for human genetic variation. *Nature*. 2015;526(7571):68-74. doi:10.1038/nature15393
e25. Schmidt AF, Finan C, Gordillo-Marañón M, et al. Genetic drug target validation using Mendelian randomisation. *Nat Commun*. 2020;11(1):3255. doi:10.1038/s41467-020-16969-0
e26. Zhu Z, Zheng Z, Zhang F, et al. Causal associations between risk factors and common diseases inferred from GWAS summary data. *Nat Commun*. 2018;9(1):224. doi:10.1038/s41467-017-02317-2
e27. Lin Z, Pan W. A robust cis-Mendelian randomization method with application to drug target discovery. *Nat Commun*. 2024;15(1):6072. doi:10.1038/s41467-024-50385-y
e28. Zuber V, Grinberg NF, Gill D, et al. Combining evidence from Mendelian randomizationMR and colocalization: Review and comparison of approaches. *Am J Hum Genet*. 2022;109(5):767-782. doi:10.1016/j.ajhg.2022.04.001

Supplementary Figures:

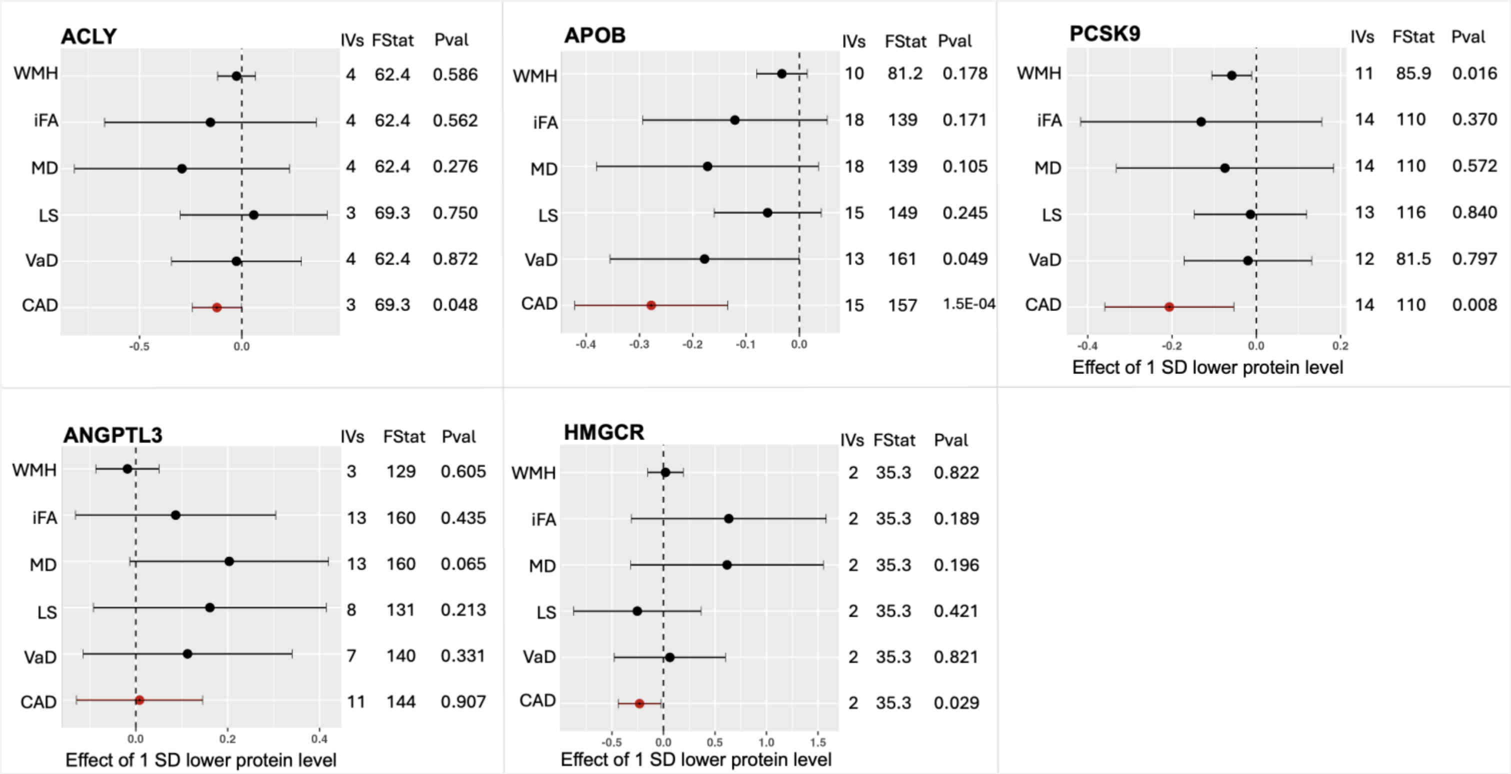

Supplementary Figure 1: Plots for Lipid-Lowering Drug Target MR using *cis* and *trans* pQTL Instruments for Analysis

Inverse variance-weighted MR (Mendelian randomization) was conducted for lipid-lowering drug targets using both cis-acting and trans-acting pQTLs (n= 35559*) as the exposure with five vascular dementia outcomes; WMH (white matter hyperintensities, n= 50559), iFA (inversed fractional anisotropy, n= 31125), MD (mean diffusivity, n= 31147), LS (lacunar stroke, , n= 6030/248929 cases/controls), VaD (vascular dementia diagnosis, n= 7008/899672 cases/controls). CAD (coronary artery disease) was used as a positive control and is indicated in red (n= 122733/424528 cases/controls). Data are presented as the causal MR estimate as the centre and error bars indicating a +/- a 95% confidence interval (CI), with the minima corresponding to the lower 95% CI bound and the maxima representing the upper 95% CI bound. The number of instrumental variables (IVs) used in each analysis is given. The F-statistic (Fstat) is also provided as a measure of instrument strength. Pval denotes uncorrected p-values; multiple-comparison corrected p-values (applied at differing levels) are available in the supplement. SD (standard deviation), pQTL (protein quantitative trait loci).*

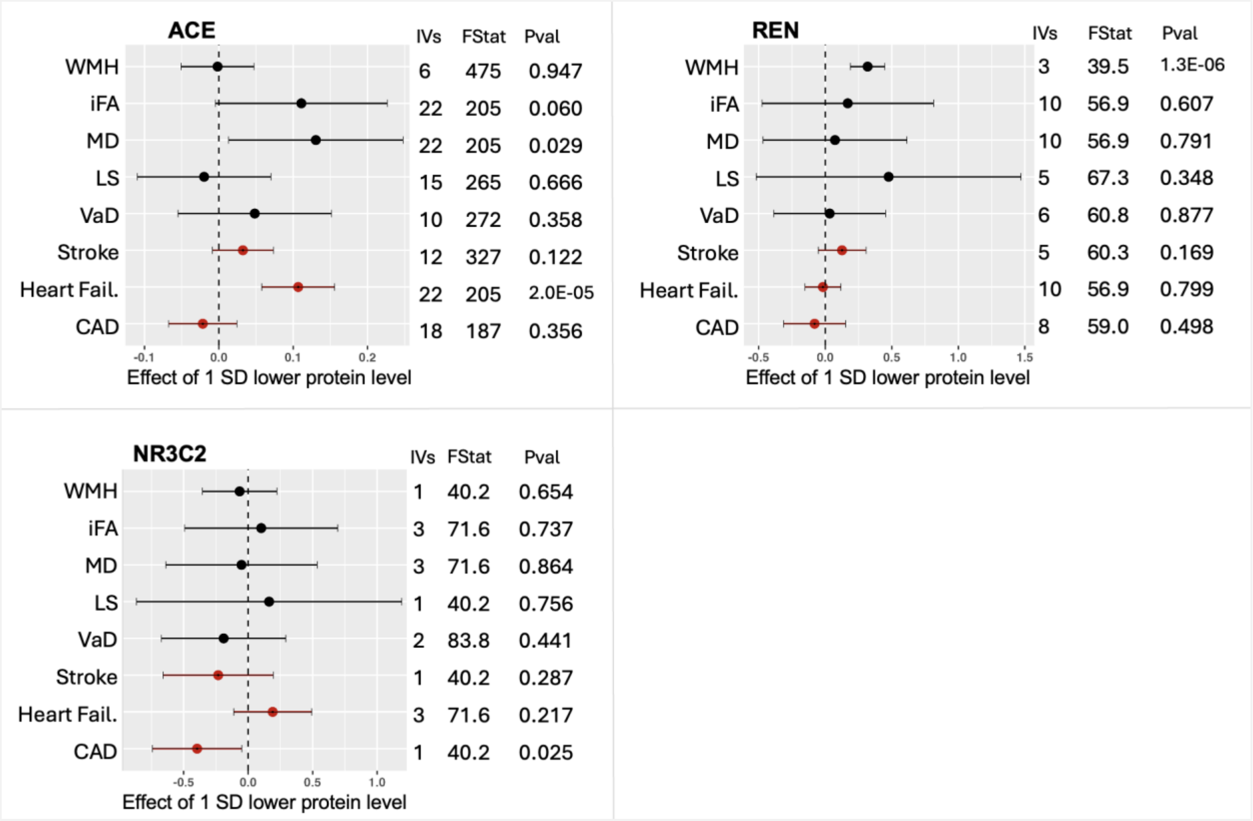

Supplementary Figure 2: Plots for Anti-hypertensive Drug Target MR using *cis* and *trans* pQTL Instruments for Analysis

Inverse variance-weighted MR (Mendelian randomization) was conducted for anti-hypertensive drug targets using cis-acting and trans-acting pQTLs (n= 35559) as the exposure with five vascular dementia outcomes; WMH (white matter hyperintensities, n= 50559), iFA (inversed fractional anisotropy, n= 31125), MD (mean diffusivity, n= 31147), LS (lacunar stroke, , n= 6030/248929 cases/controls), VaD (vascular dementia diagnosis, n= 7008/899672 cases/controls). CAD (coronary artery disease, n= 122733/424528 cases/controls), stroke (n= 67162/454450), and heart failure (Heart Fail., n= 14262/471898 cases/controls). Data are presented as the causal MR estimate as the centre and error bars indicating a +/- a 95% confidence interval (CI), with the minima corresponding to the lower 95% CI bound and the maxima representing the upper 95% CI bound. The number of instrumental variables (IVs) used in each analysis is given, for targets with multiple IVs inverse-variance weighted MR was used, for those with a single IV the Wald ratio was calculated. The F-statistic (Fstat) is also provided as a measure of instrument strength. Pval denotes uncorrected p-values; multiple-comparison corrected p-values (applied at differing levels) are available in the supplement. SD (standard deviation), pQTL (protein quantitative trait loci).

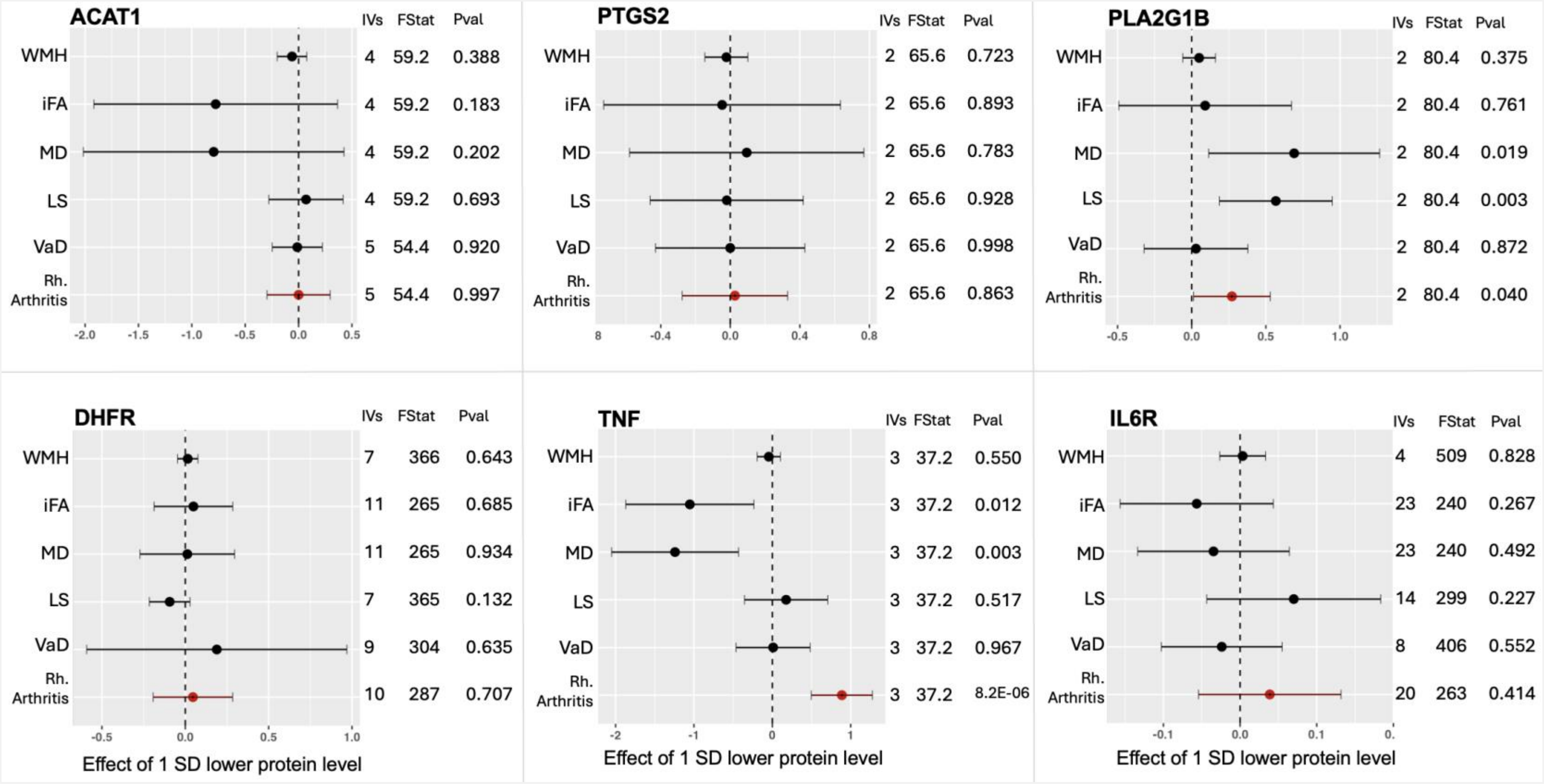

Supplementary Figure 3a: Plots for Anti-inflammatory Drug Target MR using *cis* and *trans* pQTL Instruments for Analysis

Inverse variance-weighted MR (Mendelian randomization) was conducted for anti-inflammatory drug targets using cis-acting and trans-acting pQTLs (n= 35559) as the exposure with five vascular dementia outcomes; WMH (white matter hyperintensities, n= 50559), iFA (inversed fractional anisotropy, n= 31125), MD (mean diffusivity, n= 31147), LS (lacunar stroke, , n= 6030/248929 cases/controls), VaD (vascular dementia diagnosis, n= 7008/899672 cases/controls). Rheumatoid arthritis (Rh. arthritis) was used as a positive control and is indicated in red (n= 22350/74823 cases/controls). Data are presented as the causal MR estimate as the centre and error bars indicating a +/- a 95% confidence interval (CI), with the minima corresponding to the lower 95% CI bound and the maxima representing the upper 95% CI bound. The number of instrumental variables (IVs) used in each analysis is given. The F-statistic (Fstat) is also provided as a measure of instrument strength. Pval denotes uncorrected p-values; multiple-comparison corrected p-values (applied at differing levels) are available in the supplement. SD (standard deviation), pQTL (protein quantitative trait loci).

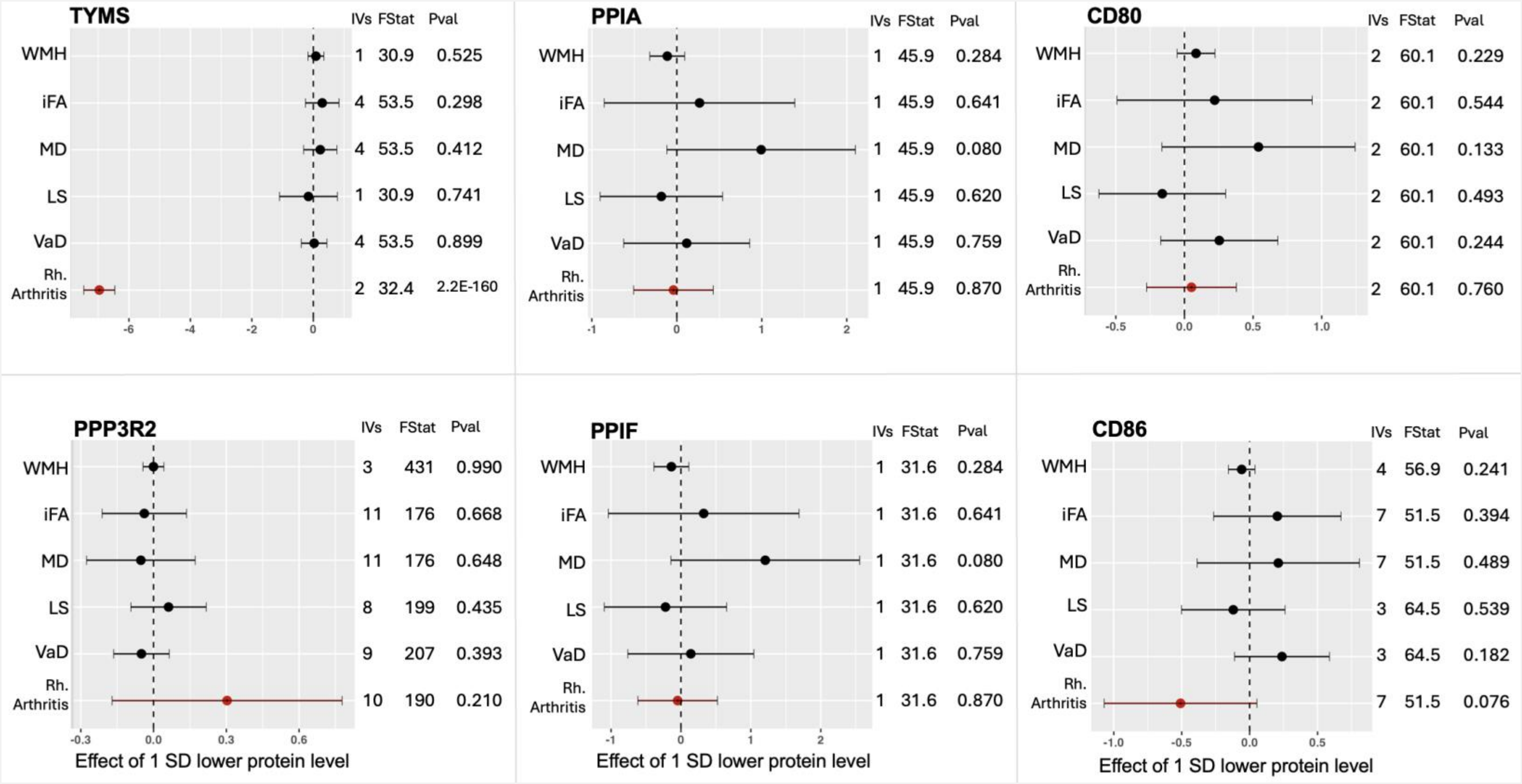

Supplementary Figure 3b: Plots for Anti-inflammatory Drug Target MR using *cis* and *trans* pQTL Instruments for Analysis

Inverse variance-weighted MR (Mendelian randomization) was conducted for anti-inflammatory drug targets using cis-acting and trans-acting pQTLs (n= 35559) as the exposure with five vascular dementia outcomes; WMH (white matter hyperintensities, n= 50559), iFA (inversed fractional anisotropy, n= 31125), MD (mean diffusivity, n= 31147), LS (lacunar stroke, , n= 6030/248929 cases/controls), VaD (vascular dementia diagnosis, n= 7008/899672 cases/controls). Rheumatoid arthritis (Rh. arthritis) was used as a positive control and is indicated in red (n= 22350/74823 cases/controls). Data are presented as the causal MR estimate as the centre and error bars indicating a +/- a 95% confidence interval (CI), with the minima corresponding to the lower 95% CI bound and the maxima representing the upper 95% CI bound. The number of instrumental variables (IVs) used in each analysis is given. The F-statistic (Fstat) is also provided as a measure of instrument strength. Pval denotes uncorrected p-values; multiple-comparison corrected p-values (applied at differing levels) are available in the supplement. SD (standard deviation), pQTL (protein quantitative trait loci).

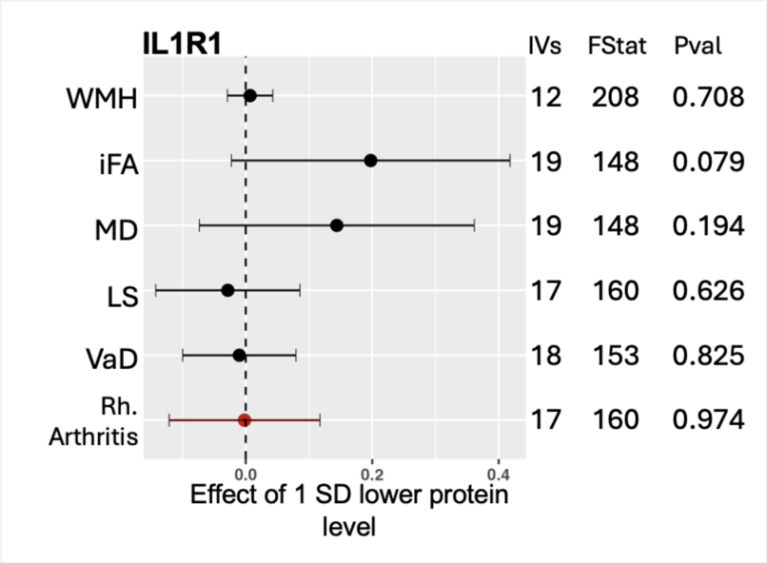

Supplementary Figure 3c: Plots for Anti-inflammatory Drug Target MR using *cis* and *trans* pQTL Instruments for Analysis

Inverse variance-weighted MR (Mendelian randomization) was conducted for anti-inflammatory drug targets using cis-acting and trans-acting pQTLs (n= 35559) as the exposure with five vascular dementia outcomes; WMH (white matter hyperintensities, n= 50559), iFA (inversed fractional anisotropy, n= 31125), MD (mean diffusivity, n= 31147), LS (lacunar stroke, , n= 6030/248929 cases/controls), VaD (vascular dementia diagnosis, n= 7008/899672 cases/controls). Rheumatoid arthritis (Rh. arthritis) was used as a positive control and is indicated in red (n= 22350/74823 cases/controls). Data are presented as the causal MR estimate as the centre and error bars indicating a +/- a 95% confidence interval (CI), with the minima corresponding to the lower 95% CI bound and the maxima representing the upper 95% CI bound. The number of instrumental variables (IVs) used in each analysis is given. The F-statistic (Fstat) is also provided as a measure of instrument strength. Pval denotes uncorrected p-values; multiple-comparison corrected p-values (applied at differing levels) are available in the supplement. SD (standard deviation), pQTL (protein quantitative trait loci).
