# Supplementary material for: Repurposing drugs for the prevention of vascular dementia using evidence from drug target Mendelian randomization
Source: Nat Aging. 2026 Apr 20;6(4):905–15. doi: 10.1038/s43587-026-01106-1 (PMC13099373; doi:10.1038/s43587-026-01106-1)
Supplement: Supplementary file 2 — Reporting Summary [file 43587_2026_1106_MOESM2_ESM.pdf]

nature portfolio

Corresponding author(s): Victoria Taylor-Bateman

Last updated by author(s): Feb 8, 2026

Reporting Summary

Nature Portfolio wishes to improve the reproducibility of the work that we publish. This form provides structure for consistency and transparency in reporting. For further information on Nature Portfolio policies, see our Editorial Policies and the Editorial Policy Checklist.

Statistics

For all statistical analyses, confirm that the following items are present in the figure legend, table legend, main text, or Methods section.

n/a	Confirmed	
☐	☒	The exact sample size (n) for each experimental group/condition, given as a discrete number and unit of measurement
☒	☐	A statement on whether measurements were taken from distinct samples or whether the same sample was measured repeatedly
☐	☒	The statistical test(s) used AND whether they are one- or two-sided<br>*Only common tests should be described solely by name; describe more complex techniques in the Methods section.*
☐	☒	A description of all covariates tested
☐	☒	A description of any assumptions or corrections, such as tests of normality and adjustment for multiple comparisons
☐	☒	A full description of the statistical parameters including central tendency (e.g. means) or other basic estimates (e.g. regression coefficient) AND variation (e.g. standard deviation) or associated estimates of uncertainty (e.g. confidence intervals)
☐	☒	For null hypothesis testing, the test statistic (e.g. F, t, r) with confidence intervals, effect sizes, degrees of freedom and P value noted<br>*Give P values as exact values whenever suitable.*
☐	☒	For Bayesian analysis, information on the choice of priors and Markov chain Monte Carlo settings
☒	☐	For hierarchical and complex designs, identification of the appropriate level for tests and full reporting of outcomes
☒	☐	Estimates of effect sizes (e.g. Cohen's d, Pearson's r), indicating how they were calculated

Our web collection on statistics for biologists contains articles on many of the points above.

Software and code

Policy information about availability of computer code

Data collection	All data was taken from publicly available data sources. The original papers for sourcing the data are provided in the manuscript.
Data analysis	Code used for analysis is provided at https://doi.org/10.5281/zenodo.15190762<br>Software used: R version 4.4.0, RStudio version 2025.05.0+496. R Packages: TwoSampleMR version 0.6.8, stringr version 1.5.1, ieugwasr version 1.0.3, coloc version 5.2.3, dplyr version 1.1.4, plyr version 1.8.9.

For manuscripts utilizing custom algorithms or software that are central to the research but not yet described in published literature, software must be made available to editors and reviewers. We strongly encourage code deposition in a community repository (e.g. GitHub). See the Nature Portfolio guidelines for submitting code & software for further information.

Data

Policy information about availability of data

All manuscripts must include a data availability statement. This statement should provide the following information, where applicable:

- Accession codes, unique identifiers, or web links for publicly available datasets
- A description of any restrictions on data availability
- For clinical datasets or third party data, please ensure that the statement adheres to our policy

All summary statistics were obtained from publicly available datasets details from the original papers as described in Table 2 and Supplementary Table 1. Links for each paper are as follows; Willer et al., 2013, Evangelou et al., 2018, Said et al., 2022, Ferkingstad et al., 2021, Sun et al., 2023, Kurki et al., 2023, MEGAVCID et al.,

2024, Sargurupremraj et al., 2020, Taylor-Bateman et al., 2022, Traylor et al., 2021, van der Harst et al., 2018, Malik et al., 2018, Sakaue et al., 2021, Ishigake et al., 2022, Folkersen et al., 2020, Vuckovic et al., 2020, de Lange et al., 2017 and the Neale Lab UK Biobank resource (http://www.nealelab.is/uk-biobank/).

Research involving human participants, their data, or biological material

Policy information about studies with human participants or human data. See also policy information about sex, gender (identity/presentation), and sexual orientation and race, ethnicity and racism.

Reporting on sex and gender	Gender was not taken into account as GWAS summary statistics were used
Reporting on race, ethnicity, or other socially relevant groupings	All data was obtained from a European population as defined by the original GWAS studies. This limitation was due to obtaining data with sufficient sample sizes for Mendelian randomization analysis and is discussed in the limitations section of the manuscript.
Population characteristics	These are provided in the original GWAS papers
Recruitment	See original GWAS papers
Ethics oversight	Only summary statistics were used in this study, no individual data was obtained. Ethics statements for each data source are provided in the original GWAS papers

Note that full information on the approval of the study protocol must also be provided in the manuscript.

Field-specific reporting

Please select the one below that is the best fit for your research. If you are not sure, read the appropriate sections before making your selection.

☒ Life sciences ☐ Behavioural & social sciences ☐ Ecological, evolutionary & environmental sciences

For a reference copy of the document with all sections, see nature.com/documents/nr-reporting-summary-flat.pdf

Life sciences study design

All studies must disclose on these points even when the disclosure is negative.

Sample size	Taken from the original GWAS source
Data exclusions	N/A
Replication	Where possible, results were replicated using pQTL data from two sources.
Randomization	Mendelian Randomization was used
Blinding	Not relevant to a MR study

Reporting for specific materials, systems and methods

We require information from authors about some types of materials, experimental systems and methods used in many studies. Here, indicate whether each material, system or method listed is relevant to your study. If you are not sure if a list item applies to your research, read the appropriate section before selecting a response.

Materials & experimental systems

n/a	Involved in the study
☒	☐ Antibodies
☒	☐ Eukaryotic cell lines
☒	☐ Palaeontology and archaeology
☒	☐ Animals and other organisms
☒	☐ Clinical data
☒	☐ Dual use research of concern
☒	☐ Plants

Methods

n/a	Involved in the study
☒	☐ ChIP-seq
☒	☐ Flow cytometry
☒	☐ MRI-based neuroimaging

Plants

Seed stocks	*Report on the source of all seed stocks or other plant material used. If applicable, state the seed stock centre and catalogue number. If plant specimens were collected from the field, describe the collection location, date and sampling procedures.*
Novel plant genotypes	*Describe the methods by which all novel plant genotypes were produced. This includes those generated by transgenic approaches, gene editing, chemical/radiation-based mutagenesis and hybridization. For transgenic lines, describe the transformation method, the number of independent lines analyzed and the generation upon which experiments were performed. For gene-edited lines, describe the editor used, the endogenous sequence targeted for editing, the targeting guide RNA sequence (if applicable) and how the editor was applied.*
Authentication	*Describe any authentication procedures for each seed stock used or novel genotype generated. Describe any experiments used to assess the effect of a mutation and, where applicable, how potential secondary effects (e.g. second site T-DNA insertions, mosiacism, off-target gene editing) were examined.*
